# Association between climate related hazards and depression among coastal communities in Indonesia

**DOI:** 10.1038/s41598-025-89298-1

**Published:** 2025-02-27

**Authors:** Asri Maharani, Sujarwoto Sujarwoto, Herni Susanti, Helen Brooks, Penny Bee

**Affiliations:** 1 Mental Health Research Group, Division of Nursing, Faculty of Biology, Medicine, and Health, Manchester Academic Health Science Centre, Midwifery and Social Work, School of Health Sciences, University of Manchester, Manchester, UK; 2 Portsmouth Brawijaya Center for Global Health, Population and Policy and Department of Public Administration, Universitas Brawijaya, Malang, Indonesia; 3 Faculty of Nursing, Universitas Indonesia, Kota Depok, Indonesia

**Keywords:** Depression, Climate-change impacts

## Abstract

Climate change has a profound impact on the mental health and well-being of people all over the world. However, studies on the impacts of climate-driven rising sea levels on mental health remain few. This study aims to examine the risk of depression among people who live in coastal areas susceptible to the natural hazards associated with climate change. We used the Indonesia Basic Health Survey 2018, which included 642,419 adults in Indonesia. Multivariable logistic regression analysis was conducted to examine the relationship between living in a coastal hazard area and depression. We included socio-demographics, health status, and health access information in the analysis to identify the most vulnerable groups. Our findings show that people who live in coastline hazard areas have 1.13 times higher odds to have depression than people who live outside those areas. Individuals living in the coastal hazards areas who were less likely to have autonomous mobility or resources, including young adults, females, those with low socio-economic conditions, and those with pre-existing health conditions, had a higher risk of depression than other groups. Culturally acceptable and effective mental health interventions should thus target these vulnerable populations and settings to effectively reduce climate-related health risks.

**Supplementary Information:**

The online version contains supplementary material available at 10.1038/s41598-025-89298-1.

## Introduction

Climate change is widely regarded as a contributing factor to a growing number of worldwide emergencies, which have a significant effect on the impacted population’s mental health and well-being^1^. Extreme weather due to climate change has caused 2.1 million deaths and globally cost $4.3 trillion in economic losses in the past five decades^2^. Evidence of the consequences of climate change on mental health and well-being has been accumulating^3,4^ Prior studies have shown that exposure to climate-related stressors, such as heat, humidity, rainfall, drought, wildfires, and floods, have been linked to increased psychiatric hospitalisations^5^, suicide rates^6^, and psychological distress^7^, in addition to worsening mental health and higher mortality among individuals with pre-existing mental health conditions^8,9^ Without action, these burdens will become greater over the coming decades.

Among the most significant consequences of climate change is rising sea levels. The global mean sea level has increased by about 20 cm since 1900^10^. The rise of sea level has been accelerating during the 20th and early 21st centuries, and it has increased at a rate of roughly 3.6 mm every year from 2006 to 2015^11^. Over 600 million people worldwide reside in low-lying coastal regions at a height of less than 10 m. By 2050, the number of people living in these areas is anticipated to surpass 1 billion^12^. Sea level rise is predicted to exacerbate coastal erosion, catastrophic marine flooding, and saltwater intrusion in coastal aquifers^13^. It has further heightened the impact of hurricanes in coastal areas^14^. Sea level rise increases the risk of many forms of bodily harm: injury or drowning when coupled with extreme weather, infectious diseases from increased exposure to waterborne or vector-borne pathogens, health effects from increased exposure to contaminants or airborne pollutants, and numerous negative effects on social determinants of health. It is thus also important to understand the current and future mental health consequences of sea level rise in those communities most vulnerable to it. In this study, we examine areas affected by sea level rise to enable the development of targeted resilience building and intersectoral mitigation strategies.

Recent studies have begun responding to the challenges presented by sea level rise in affected locations. In a study in the Solomon Islands, almost all (56 out of 57) respondents stated that they and their families were being impacted by sea level rise, which was creating anxiety and panic both personally and throughout the community^15^. Kelman et al. (2020) reported that populations in small island developing states experienced major adverse effects on mental health and well-being linked to climate change, including acute stress, anxiety, depression, and post-traumatic stress disorder (PTSD)^16^. A study in two counties in Florida, USA, revealed that sea level rise and tropical cyclones were associated with a high risk of PTSD, anxiety, and major depressive disorder. However, these studies focused on limited geographical areas or selected islands and atolls and used community or volunteer samples rather than national ones. Hence, their findings cannot be widely generalised.

This study addresses the above-mentioned gap by combining two nationally representative datasets from Indonesia. As the world’s largest archipelagic country, Indonesia’s coastal area is vulnerable to climate change. Indonesia ranks fifth in the world in terms of residents living in lower-elevation coastal zones, and without adaptation, the total population at risk of permanent flooding by 2070–2100 could exceed 4.2 million people^17^. Climate change has altered the characteristics of Indonesia’s coastline, including a decline in the natural coastline and an increase in the artificial coastline^18^. Over the last fifteen years, Indonesia has lost 29,261 hectares of its coastline—an area about the size of Jakarta—while natural sedimentation creates 895 hectares of new coastal land annually. According to a Ministry of Maritime Affairs and Fisheries report, erosion is causing Indonesia to lose some 1,950 hectares of coastal land annually^19^.

Furthermore, floods, the possibility of irreversible flooding, and the incursion of saltwater into freshwater resources could render other locations uninhabitable^20^. Prior studies have revealed the increasing health risks, including vector-borne diseases, tuberculosis, diarrhoea, and skin diseases, due to climate change among coastal populations in two areas in Indonesia: Semarang^21^ and Manado^22^. Certain groups are more vulnerable to the health effects of climate change than others because of social and economic factors such as income, education, access to health care, and housing. Identifying these groups is crucial in designing interventions to tackle the burden of mental disorders in areas affected by climate change.

While prior studies have identified physical health risks due to climate change in Indonesia’s coastal populations, mental health impacts remain underexplored. Mental health infrastructure in the country presents both opportunities and challenges in addressing these emerging needs. Mental health has historically been a lower priority within the national health agenda, with limited funding and infrastructure concentrated in urban areas, leaving coastal and rural regions underserved^23^. Only 6% of Indonesia’s national health budget is allocated to mental health, with mental health staff ratios significantly below the global average of 9 per 100,000 population^24^. The government launched the Mental Health Act in 2014^25^, yet Indonesia has an estimated 0.31 psychiatrists, 2.52 mental health nurses and 0.17 psychologists per 100,000 people, and mental health facilities are scarce, particularly in remote and coastal regions. In comparison, this ratio varies widely worldwide, from fewer than 2 per 100,000 in low-income countries to 70 per 100,000 in high-income countries. Barriers to mental health care access include stigma, insufficient awareness, and logistical challenges, such as transportation to distant facilities. Nonetheless, recent government and non-governmental and community-led initiatives have begun addressing these gaps by promoting mental health awareness and developing localised interventions. Expanding mental health access for climate-affected populations will require integrating culturally appropriate programs into Indonesia’s public health strategy, prioritising the most vulnerable and underserved communities.

Given these challenges and the limited research on the mental health impacts of climate-driven coastal hazards in Indonesia, this study seeks to address this gap by exploring the mental health burden associated with living in vulnerable coastal areas. The specific objectives of this study are:

1. Examine the consequences of living in areas affected by coastal hazards on the risk of depression.
2. Evaluate the effects of coastal hazards, i.e., abrasions, hurricanes, and tidal flooding, on depression.
3. Identify groups within the population living in areas with coastal hazards at higher risk of depression.

## Study design and method

### Study design

This study used a cross-sectional design with data from the most recent Indonesia Basic Health Survey (*Riset Kesehatan Dasar* or *Riskesdas*) 2018^26^. Riskesdas is a nationally representative survey conducted every five years in all 34 provinces and 514 districts of Indonesia by the National Institute of Health Research and Development (NIHRD), Ministry of Health; it focuses on the measurement of health indicators mandated by the Millennium Development Goals or Sustainable Development Goals, including mental health and non-communicable diseases.

The Ethics Committee of the NIHRD provided ethical clearance before data collection. Because we conducted secondary data analyses, the pre-requisite of ethics approval is not applicable. Prior to the data collection and before the interviews were conducted, the enumerators obtained informed consent from the respondents in the form of written consent.

Participants were selected using a multistage systematic random sampling method. The first stage identified groups of census blocks and designated them as primary sampling units (PSUs). The second stage used a probability proportional sampling method to design the enrolment size to identify a census block from each PSU. The third comprised systematic random sampling of 25 census buildings from each block. One household from each census building was randomly chosen in the fourth stage. All household members (defined as those having stayed on the premises for the past six months or more and having the same financial source for food) of each selected household were asked to participate in the survey. The study sample focuses on adults aged 18 years and older who had completed information on mental health depression questions. The total number of respondents whose information was used in the analyses was 642,419.

The Riskesdas 2018 data was linked to the Village Survey (*Potensi Desa* or Podes) data in the same year (2018). Podes provides information on potential assets that belong to the smallest area unit in Indonesia, i.e. the village, including its social economy, infrastructure, and human resources. The Podes data provides information at the village level (desa), which we aggregated to the district level (kabupaten/kota). Similarly, the Riskesdas data is available at the individual level but includes identifiers for the district level (kabupaten/kota). For this study, we retrieved information from Podes 2018 on whether the village is situated in a coastal district and has experienced abrasion, hurricanes, and/or tidal flooding in the years 2015 to 2017. We aggregated Podes data at the district level using village and district identifiers provided by Statistics Indonesia. Specifically, we summarised data on the occurrences of coastal abrasions, hurricanes, and tidal flooding at the village level, then aggregated these counts within each district. The Podes data is reported by village governments rather than geophysical experts, with the occurrence of coastal hazards based on reports provided by village leaders within their respective villages. After this aggregation, we merged the district-level data with individual data from the 2018 Riskesdas survey, using district codes from Statistics Indonesia to ensure accurate alignment. Taken together, these data capture the nested structure of individuals within districts.

Ethical approval for Riskesdas was obtained via the Ethical Commission of the National Institute of Health Research and Development, Ministry of Health, the Republic of Indonesia (No. LB.02.01/2/KE.267/2017). Participants gave informed consent to participate in the study before taking part. Procedures were performed in accordance with national guidelines and regulations for research activities. We did not conduct the data collection ourselves; rather, we analysed data collected by the Riskesdas team using a standardised multistage sampling method to ensure national representation. Our analysis focuses on adult respondents aged 18 years and older who completed the mental health depression questions.

### Measures of depression

Depression was assessed using the Mini-International Neuropsychiatric Interview (MINI) version 6 of the Diagnostic and Statistical Manual of Mental Disorders (DSM)-IV and the International Classification of Diseases − 10, a structured diagnostic psychiatric interview used to assess various mental health problems^27–29^. It is widely used in clinical and research settings and has been translated into various languages. A previous study validated the Indonesian version of the MINI depression Sect^30^. The questionnaire consists of ten questions with “yes” or “no” answers. A respondent was classed as depressed if they answered “yes” to at least two of the questions numbered 1 to 3 and “yes” to at least two of the questions numbered 4 to 10.

### Measures of coastal hazards

We categorised the district as a coastal district if it has villages immediately on the coastline. The information on the coastal hazards was gained from Podes 2018. It asked about the presence and the number of coastal abrasions, hurricanes, and tidal flooding in 2015, 2016 and 2017. Events across years were added together. We classified the respondents as living in a district with coastal abrasion, living on a coastline with hurricanes, and having regular experience with tidal flooding if they lived in a district having experienced those disasters from 2015 to 2017.

### Covariates

We classified individual-level risk factors of depression as demographic, socio-economic, health behaviour-related, health status-related, and household characteristic-related. The demographic factors included age group (18–24 as reference, 25–34, 35–44, 45–54, 55–64, 65–74, 75+) and sex (male as reference). Marital status was categorised as single (reference), married, or divorced/widowed. The socio-economic factors included the highest attained educational level (primary school or less, junior high school, and senior high school and higher as reference), household expenditure per month in quintiles, and occupation (unemployed as reference, student, employed/retired, self-employed, informal worker, and other).

Smoking, alcohol use and physical activity were included as indicators of health behaviour determinants. We categorised smoking behaviour into smoking every day (reference), not every day, past smoker and non-smoker. Similarly, alcohol use was classified as drinking alcohol under standard (reference), more than standard, or never drinking alcohol. The presence of chronic diseases was measured using self-reports of ever having been diagnosed by a health professional as suffering from tuberculosis, hypertension, stroke, diabetes mellitus, heart disease, asthma, rheumatoid arthritis, cancer, or renal failure. We used Principal Component Analysis (PCA) to create an index of healthcare access difficulty, based on three variables: (1) the modes of transportation used to access healthcare services, (2) the round-trip time to reach the nearest healthcare provider, and (3) the transportation cost for a round trip^31^. PCA was applied to these variables to capture the underlying dimension of ‘difficulty in accessing healthcare’ by summarising them into a single index. This approach reduces dimensionality and allows us to represent access difficulty in a way that combines multiple related factors. We retained the first principal component, which accounted for the largest proportion of variance among these variables, as the difficulty index for healthcare access. This index was then dichotomised around the mean/median to classify respondents as having either ‘difficult’ or ‘easy’ access to healthcare services.

### Statistical analyses

We analysed the data in several steps. Firstly, we created maps to illustrate the importance of residential areas with regard to coastal hazards and the prevalence of depression across 514 districts in Indonesia. Secondly, we described the characteristics of the respondents in terms of residential area and the presence of depression. For the bivariate analysis, continuous variables between different sex and rurality were compared using t-test, while categorical variables were compared using chi-square tests. We presented the odds ratios (ORs) and significance of the difference by implementing the alpha of 5% error through χ2 analysis for the covariates with two categories and binary logistic regression for the covariates with more than two categories. Finally, multivariable logistic regression analysis was conducted to examine the relationship between living in a coastal hazard district and the risk of depression. We performed the multivariable logistic regression for all samples and respondents living in districts with all three types of coastal hazards combined and separately for those living in districts with abrasion, hurricanes, and tidal floods. We calculated marginal effects to estimate the predicted probability of having depression with healthcare access and household expenditure as outcome variables. We used STATA 18.0 for this analysis, considering the corresponding Riskesdas weights, strata, and primary sampling unit according to its survey design. Marginal effects were calculated to estimate the predicted probability of depression associated with each independent variable, controlling for other covariates. Specifically, after running the multivariable logistic regression models, we computed the marginal effects at the mean values of the covariates. This approach allows for an interpretation of how changes in variables, such as healthcare access or household expenditure, affect the likelihood of depression while holding other factors constant. The calculations were performed using the *margins* command in STATA 18.0^32^, providing an average effect across the sample that reflects the probability difference associated with each predictor variable. We further applied survey weights provided by the Riskesdas dataset to account for the complex sampling design and ensure that our results nationally represent the Indonesian adult population. Riskesdas uses a multistage sampling approach to select participants, which may result in unequal selection probabilities across different population groups. The weights adjust for these differences, making our estimates more reflective of the broader population. All analyses, including descriptive statistics, logistic regression, and marginal effects calculations, were weighted using *svy* commands in STATA 18.0^33^, allowing for accurate estimation of survey data with complex sampling designs. Finally, the Bonferroni adjustment was used (p value < 0.01) for analysing all regression associations in order to control for Type 1 error.

For the robustness check, we provided the analysis using four different models. Model 1 was adjusted with demographic (age and sex) and socioeconomics variables (marital status, employment status, and household expenditure); Model 2 was adjusted with demographic, socioeconomic, and lifestyle variables; Model 3 was adjusted with demographic, socioeconomic, lifestyle (smoking status, alcohol consumption and physical activity) and the presence of comorbidities variables; and Model 4 was adjusted with demographic, socioeconomic, lifestyle, the presence of comorbidities and access to healthcare variables.

## Results

### Geographical distribution of SLR and depression

**Fig. 1:**
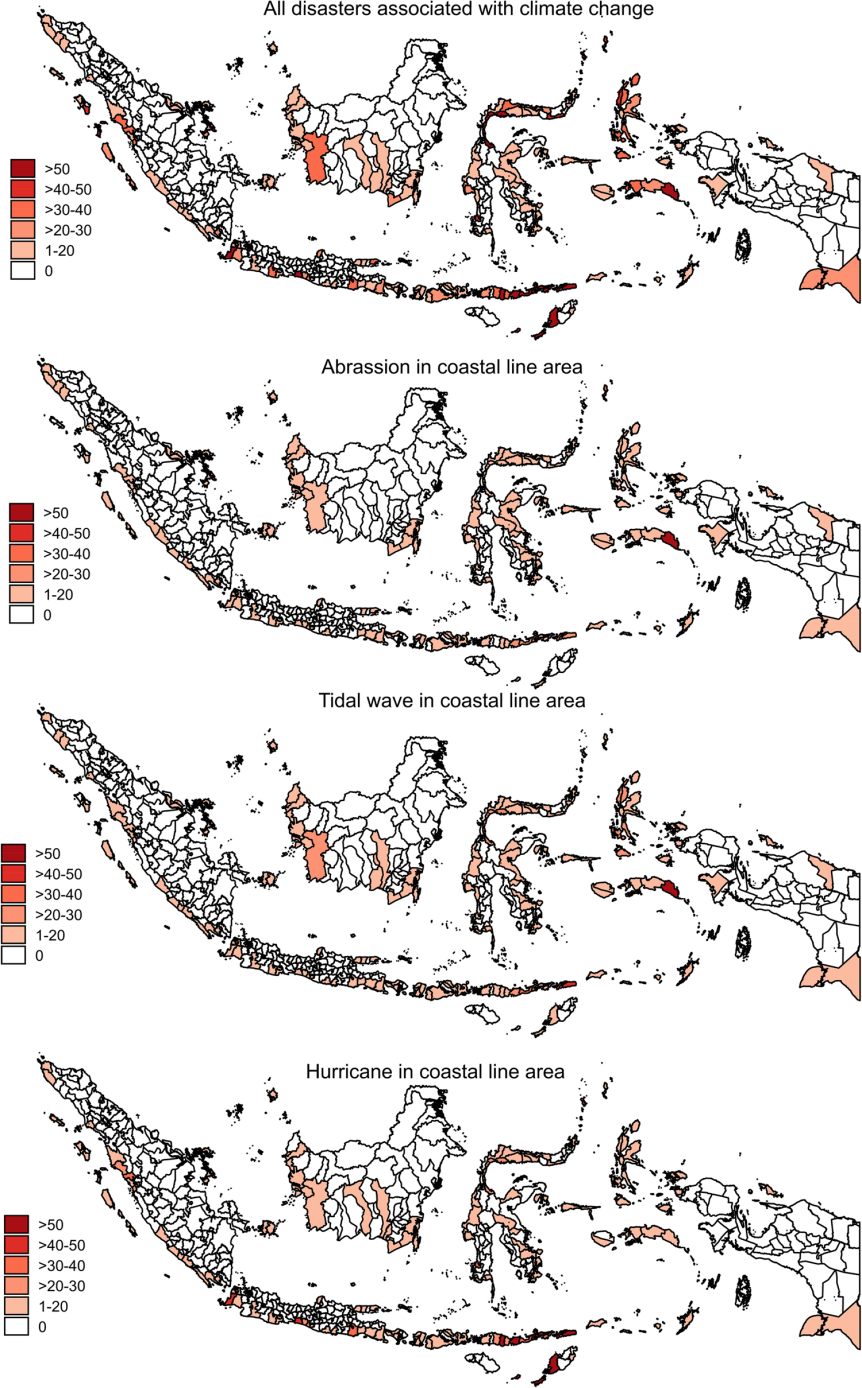
Geographical distribution of the number of coastal hazards, abrasions, hurricanes and tidal floodings, among districts in Indonesia 2016–2018. *Source*: Podes 2018.

Figure 1 describes the geographical distribution of abrasion, hurricanes, and tidal flooding across districts in Indonesia. The map shows the variations in occurrence of these natural hazards, with the highest number found across the northern and southern Java coastlines, northeastern and southern Sumatra coastlines, West Sumatra coastline, northwest Sulawesi coastline, southern Papua coastline, and on the small islands of southern Maluku and eastern Nusa Tenggara. More than one hundred of the coastline hazard areas are found on those islands.

**Fig. 2:**
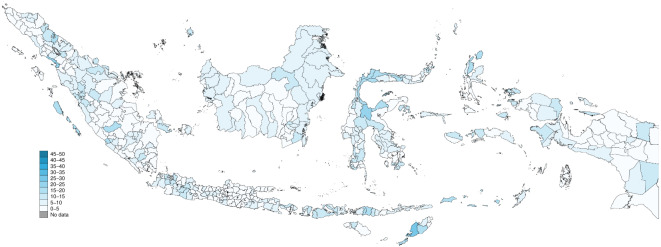
Geographical distribution of depression prevalence in (%) among districts in Indonesia 2018. *Source*: Riskesdas 2018.

The geographical variation in the prevalence of depression across districts is also apparent (Fig. 2). People living across the northern and southern Java coastlines, northeastern Sumatra, West Sumatra and the southeastern Sumatra coastline, northwestern Sulawesi coastline, southern Papua coastline, and on the small islands of southern Maluku and eastern Nusa Tenggara had the highest prevalence of depression with 20–30% of their populations having depression.

### Characteristics of respondents

The first four columns of Table 1 describe the characteristics of respondents’ socio-demographic and depression status based on living area (coastal or non-coastal). A detailed description of respondents based on types of coastal hazards (abrasion, hurricane, and tidal flood exposure) is presented in Supplementary Tables S1 and S2.

The percentage of respondents who lived in coastal hazard districts was 31.3%. The prevalence of depression among respondents living in coastal hazard districts (6.7%) was higher than among those living outside coastal hazard districts (5.7%). The highest prevalence of depression was found among people living with tidal flood exposure (7.5%). The age distributions in the two types of districts were relatively similar, with 23.5% being in the 35–44 age group. In both types of districts, the proportion of females was slightly higher than males. The percentage of respondents educated at the primary school level or less was higher among those living in coastal hazard districts (48.8%) than those living outside (43.5%). The highest proportion of people with a low educational level was found among those living in tidal flood districts (51.4%). Likewise, the percentage of respondents working in informal sectors within coastal hazard districts was higher (37.4%) than those living outside (33.8%). The highest percentage of informal workers was found among people living in tidal flood districts (40.0%). The proportion of families within the first quintile of expenditures within coastal hazard districts was also larger (20.9%) than those outside (16.4%). The highest percentage of low-income families was located in coastal districts frequently facing tidal floods (23.0%). People living in coastal hazard districts had less access to healthcare than their counterparts, as 14.1% of respondents living in those districts reported difficulty accessing healthcare compared with 10.1% of those living outside them. The highest percentage of respondents facing difficulty accessing healthcare appeared in coastal districts frequently facing tidal floods (15.8%).

Smoking was slightly more prevalent among people living in coastal hazard districts (27.2%) than 26.8% of those living outside coastal hazards districts. The highest percentage of smokers was found among people living in tidal flood districts (28.5%). Likewise, the proportions of those using alcohol both under and above standard were larger among people living in coastal hazard districts (3.3% and 3.2%, respectively) than those living outside coastal hazard districts (2.5% and 1.4%, respectively). People living in coastline districts were also less likely to engage in physical activity (10.1%) than those living outside coastline districts (9.8%). The highest percentage of people with less physical activity was found among people living in tidal flood districts (9.4%). The most common illnesses among people living in coastal hazard districts were hypertension (8.7%) and rheumatoid arthritis (8.0%), while the least common was cancer (0.3%).

The last three columns of Table 1 show the characteristics of respondents living in districts experiencing coastal hazards with respect to the presence of depression. We found the highest percentage of depression among respondents living in coastal hazard districts within the 35–44 age group (22.1%), females (62.7%), those with primary school or less education (69.7%), informal workers (37.4%) and unemployed people (37.0%), those in the first quintile of expenditures (25.5%), and those with difficulty to access healthcare (20.8%). These socio-demographic characteristics were similar among people living in areas where abrasion, hurricanes, and tidal flooding often occur, with a slightly higher percentage found among people living in tidal flooding districts.

About one-fifth of respondents living in coastal hazard areas with depression smoked every day (23.4%). The highest percentage of smokers was found among depressed people living in tidal flooding districts (24.6%). The percentage of alcohol use was higher among respondents living in coastal hazard districts with depression than those without depression. One-tenth of these reported having little physical activity. Among depressed people, 14.7% had rheumatoid arthritis, and 14.2% had hypertension.

### Multivariable logistic regression

Table 2 describes the results of the multivariable logistic regression. Respondents living in coastal hazard districts had 1.13 times the odds of experiencing depression than those living outside them. Likewise, respondents living in areas with coastal abrasions had 1.16 times higher odds of experiencing depression than those living outside those districts. Those living in coastal districts with hurricanes had 1.14 times higher odds of experiencing depression than their counterparts. Those living in coastal districts with tidal flooding had 1.24 times higher odds of experiencing depression. In all models, young adults (18–24 years) had higher odds of depression than members of other age groups. Females have 2.16–2.17 times higher odds of experiencing depression than males. Those who graduated from junior high school and those with a primary school education or less had higher odds of having depression than those who graduated from senior high school. Married people had higher odds of experiencing depression than single people. The odds of being depressed for divorced and widowed individuals were 1.12–1.13 times greater than those of single individuals. In all models, jobless individuals had higher odds of experiencing depression than employed individuals.

The association of household expenditures with the odds of depression varied across respondent categories. In all models, the 2nd and 3rd quintile of expenditure was not significantly associated with depression. In these models, households in the 4th and 5th quintiles had lower odds of experiencing depression than those in the 1st quintile of expenditures. In all models, past smokers had higher odds of experiencing depression than regular smokers (OR = 1.24–1.25). In contrast, non-smokers had lower odds of experiencing depression than regular smokers (OR = 0.62). Those who reported not drinking alcohol had lower odds of experiencing depression than those who did consume alcohol. Respondents who engaged in physical activity had lower odds of experiencing depression than those who were less active. In all models, individuals with non-communicable diseases (lung tuberculosis, hypertension, stroke, diabetes mellitus, heart disease, asthma, rheumatoid arthritis, cancer, and kidney failure) had higher odds of experiencing depression than those without these illnesses. People with difficulty accessing healthcare had higher odds of experiencing depression (OR = 1.37).

Table 3 describes the results of multivariable logistic regression for respondents living in coastal hazard districts in general and those living in coastal abrasion districts, coastal hurricane districts, and tidal flooding districts. No significant association with depression was found in the 35–44 age group, but other age groups showed a significant association with depression. The results highlight that young adult (18–24 years) had higher odds of suffering from depression. In all categories of respondents living in coastal hazard districts, females had higher odds of experiencing depression than males. Those educated at the junior secondary level and those with a primary school education or less had lower odds of experiencing depression than those educated at the senior high school level or higher. Divorced and widowed respondents had 1.14 higher odds of experiencing depression than unmarried respondents among those living in districts with coastal hazards. Married respondents had lower odds of experiencing depression. In all categories of respondents living in coastal hazard districts, jobless respondents had higher odds of experiencing depression than employed and retired respondents.

The association of household expenditure with depression varied across the categories of residential areas. Within coastal abrasion and tidal flooding districts, significant associations were found within the 4th and 5th quintiles. The findings show that households within the poorest quintiles had higher odds of experiencing depression. In all categories of residential areas, ex-smokers had higher odds of experiencing to have depression than regular smokers (OR = 1.15–1.24). In contrast, non-smokers had lower odds of experiencing depression than regular smokers (OR = 0.66–0.68). Those who reported not drinking alcohol had lower odds of experiencing depression than those who consumed alcohol. Respondents who were physically active had lower odds of experiencing depression than those who were less active (OR = 0.90–0.93). In all models, individuals with lung tuberculosis, hypertension, stroke, diabetes mellitus, heart disease, asthma, rheumatoid arthritis, cancer, and kidney failure had lower odds of experiencing depression than those without these illnesses. The greatest odds of depression in the illness category were found among respondents who reported having cancer (OR = 2.63). People with difficulty accessing healthcare had also higher odds of experiencing depression (OR = 1.41–1.45). Supplementary Tables 3 to 7 provided the multivariate logistic models for each coastal hazard variable. They show that the significant associations between coastal hazard and odds of depressions remain and slightly diminish with the additions of different covariates.

### Marginal effects of healthcare access and household expenditure

Marginal effect analyses were employed to examine the predicted probability of having depression with healthcare access and household expenditure as outcome variables. Supplementary Fig. 1 shows the marginal effect of healthcare access based on each type of coastal hazard. Controlled for socio-demographic characteristics and types of illness, respondents who had difficulty accessing healthcare had a higher probability of experiencing to have depression than those with less difficulty accessing healthcare. Supplementary Fig. 2 describes the marginal effects of household expenditure based on each type of coastal hazard. The results highlight that individuals belonging to the poorest households had a higher probability to have depression.

## Discussion

This study is among the first to address the effect of sea level rise on depression using nationally representative data from Indonesia. Nearly one-third of the respondents to the survey lived in districts affected by coastal hazards, and the prevalence of depression was higher among them (6.7%) than among those not affected by coastal hazards (5.7%). Living in a coastal hazard district is correlated with 1.13 times higher odds of having depression. This finding supports the hypothesis that climate change-induced events can impact mental health, consistent with existing evidence of the mental health impacts from rising sea levels in the Solomon Islands^15^, the United States^34^ and the Pacific Islands^35^. Using data from two coastal counties in the US, Monsour and colleagues found that individuals affected by tropical cyclones were at higher risk of major depressive disorders (coefficients between 2.2 and 2.8)^34^. A study in the Pacific Islands interviewed 100 Tuvalian participants and revealed that 62% of them experienced psychological distress^35^. Asugeni and colleagues interviewed 57 individuals living in a remote coastal region of the Solomon Islands. They showed that 90% of them stated that they feared and worried about the impact of sea level rise^15^.

Sea level rise may affect mental health directly by exposing people to trauma. Rising sea levels cause shoreline abrasion^36^ and escalate the risk of coastal flooding^37^. Our findings showed that individuals living in districts with coastal abrasion, hurricanes, and tidal flooding were 1.16, 1.14, and 1.24 times had higher odds of experiencing depression than those living outside these districts. A prior study using data from the English National Study of Flooding and Health revealed that flooding was associated with 7.77, 4.16 and 14.70 higher odds of depression, anxiety and PTSD, respectively^38^. The risks of depression among people affected by extreme events in our study are lower than the study from the UK, probably due to the lower prevalence of depression in the national survey in Indonesia used in this study (6%) compared to the prevalence of depression in the UK (16%)^39^. Depression and anxiety scores among individuals affected by seasonal floods in Northern India have also been found to be higher than those not affected^40^. The effects of hurricanes on mental health have been documented in several studies^41,42^ For example, Kohn assessed PTSD and major depressive disorder in hurricane-affected adults at two months and two years after the event^41^. The study found that the prevalence of both diseases has remained generally steady over time (PTSD 10.6% and 11.8%, major depressive disorder 19.5% and 19.4% at two months and two years). The impact of hurricanes has also been found to be persistent in New York City and Long Island residents^42^. Having depression and anxiety immediately after a hurricane was a predictor of persistent depression and anxiety a year later.

Coastal hazards also lead to the physical loss of land and dwellings, food and water shortages, loss of employment, and eventual displacement, including migration. Munro and colleagues found a strong link between displacement following the 2013-14 floods in the UK and the prevalence of depression, anxiety, and PTSD one year later^43^. A link between displacement from one’s home after a climate-related disaster and growing mental health symptoms has also been found in Bangladesh^44^. Coastal hazards can also affect mental health indirectly by affecting physical health and community well-being. Sea level rise is associated with increased exposure to waterborne pathogens, vector-borne diseases, saltwater intrusion, and poor air quality due to mould^45^. For example, the sea level change had a positive and significant association with a higher prevalence of dengue disease in Malaysia^46^. Poor physical health may thus increase the risk of depression among people living in coastal communities.

Our findings further identify that women, those with low educational attainment, informal workers, those with lower incomes, those with comorbidities and those with difficulties accessing healthcare were among the groups with a higher odds of experiencing depression when exposed to coastal hazards. Women may be at increased risk of depression due to gender-differentiated social roles and a lack of access to resources. A study in coastal Bangladesh looked at Hurricane Aila’s effects in 2009 and found that women could not attend non-governmental organisation (NGO) training or income-generating activities without their husbands’ approval due to gender roles. The researchers also discovered that labouring in the fields in the rising heat, extracting water from seawater-contaminated wells, and damaged infrastructure by recurring tidal flooding made it more difficult for women to obtain everyday resources, which led to increased levels of hunger^47^. Rising sea levels inundate coastal areas, swallowing up agricultural land in Indonesia. This land loss directly impacts food production, especially rice, a vital crop for Indonesia. Studies estimate millions of hectares of farmland could be lost^48^. A review showed that women suffer more detrimental impacts of climate change because of social and cultural norms regarding gender, such as women tend to have less power in decision-making in the family, as well as a lack of access to and control over assets, with some exceptions^49^. Literature has highlighted that poverty is a major factor influencing people’s vulnerability to climate-related shocks and stressors^50^, and thus may be at higher risk of mental disorders. According to a study in Bangladesh, having an unpaid job or suffering considerable income loss during Cyclone Amphan was significantly associated with higher psychological distress symptoms^44^. Climate-related disasters, including sea level rise, may destroy crops, land, and critical infrastructure. These lead to reduced agricultural output and increased prices of major crops, greatly impacting food production and threatening food security^51^. Lower output of crops means lower incomes for the most vulnerable people. Under these conditions, the poorest people will be those most affected by sea level rise as they already use most of their incomes for food and require additional income to meet their daily nutritional requirements. Several South Asian countries, including Bangladesh, India, Pakistan and Nepal, launched cash transfer programs to help low-income families cope with climate-related disasters and income losses^52^. Indonesia’s government also provides a cash transfer program for poor people called Direct Cash Assistance (*Bantuan Langsung Tunai*) program. However, little is known regarding the effectiveness of this program in helping poor communities cope with climate change.

There are some limitations to this study that should be noted. First, our study is cross-sectional, which implies important limitations to the interpretability of data in terms of finding causal effects between variables. Second, the only measure available is depression. More severe mental disorders, including PTSD, acute stress disorders, anxiety, substance use and suicide, may occur as a result of the impact of climate change-related disasters^3^. Third, limitation of this study is our reliance on data from 2015 to 2017 as reported in PODES 2018 for assessing coastal hazard exposure. The available dataset focuses on the presence and frequency of coastal abrasions, hurricanes, and tidal flooding within this three-year period, thus restricting our analysis to more recent events. However, prior research has demonstrated that mental health impacts of natural hazards often emerge within months to a few years of the event, such as the 11 and 28 month follow-up period used by Schwartz et al. following Hurricane Sandy^42^ and the two-year follow-up reported by Kohn et al. after Hurricane Mitch^41^. As a result, while our findings provide meaningful insights into the effects of coastal hazards in the past three years on mental health, they may underestimate longer-term impacts. Future studies could benefit from incorporating more extended timelines or examining cumulative exposure effects to address these potential limitations. Finally, our data was taken in 2018 and needs to be updated with the rapid changes in the environment due to climate change. Future studies, including more mental disorders, could better capture the consequences of sea level rise on mental health. Targeted coproduced qualitative works are also required to integrate and refine the mechanisms of action and most modifiable intervention points.

In conclusion, living in areas affected by coastal hazards in Indonesia is related to higher odds of having depression, and almost one-third of the Indonesian population (31.3%) live in these areas. It is thus important to implement interventions to mitigate the impact of sea level rise on mental health and well-being. So far, the available adaptation strategies for sea level rise have been focused on preventing land damage, such as building sea walls, reforestation, upgrading existing drainage infrastructure, and preventing socio-economic damage due to the loss of land^53,54^ Our findings highlight the importance of further research on interventions to address the impact of rising sea levels on mental health. As mental health remains under-resourced in Indonesia, culturally appropriate approaches that engage community leaders, utilise digital health tools, and leverage local resources may be effective in overcoming access barriers^55,56^ Collaborations with non-governmental organisations and community groups could also facilitate awareness campaigns to reduce stigma and encourage early help-seeking behaviours. Based on our findings, women, those with informal jobs, low education, low income, comorbidities, and difficulty accessing healthcare have higher odds of being affected by sea level rise. Given the established challenges in accessing mental health care, policies supporting mobile mental health units, telemedicine, and community-based psychosocial support could provide much-needed care for these vulnerable populations. In addition, pre-existing health burdens make people more vulnerable to sea-level effects by reducing resources. Therefore, we need to continue to emphasise investment in mental health and non-communicable disease management to ensure resilience in this impending context. Adaptation strategies and mental health interventions should thus target these groups.

**Table 1:** Characteristics of all respondents based on residential area (with or without coastal hazards) and of respondents living in coastal hazard areas by presence of depression and the bivariate analyses. *Source*: Riskesdas 2018 and Podes 2018.

Variables	All respondents			Respondents living in coastal hazard districts (*N* = 201,109)		
	Non-coastal hazard districts (*N* = 441,310)	Coastal hazard districts (*N* = 201,109)	*p*-value*	Not depressed (*N* = 187,548)	Depressed (*N* = 13,561)	*p*-value*
Depression						
No	416,256 (94.3%)	187,548 (93.3%)	< 0.001			
Yes	25,054 (5.7%)	13,561 (6.7%)				
Age						
18–24 years old	59,746 (13.5%)	26,962 (13.4%)	< 0.001	25,133 (13.4%)	1829 (13.5%)	< 0.001
25–34 years old	90,473 (20.5%)	41,953 (20.9%)		39,616 (21.1%)	2337 (17.2%)	
35–44 years old	104,336 (23.6%)	47,358 (23.5%)		44,361 (23.7%)	2997 (22.1%)	
45–54 years old	88,343 (20.0%)	39,594 (19.7%)		36,818 (19.6%)	2776 (20.5%)	
55–64 years old	58,999 (13.4%)	26,188 (13.0%)		24,269 (12.9%)	1919 (14.2%)	
65–74 years old	26,677 (6.0%)	12,909 (6.4%)		11,808 (6.3%)	1101 (8.1%)	
75 years old and above	12,736 (2.9%)	6145 (3.1%)		5,543 (3.0%)	602 (4.4%)	
Sex						
Male	210,023 (47.6%)	94,678 (47.1%)	< 0.001	89,618 (47.8%)	5060 (37.3%)	< 0.001
Female	231,287 (52.4%)	106,431 (52.9%)		97,930 (52.2%)	8,501 (62.7%)	
Education						
Senior high school or higher	169,555 (38.4%)	70,420 (35.0%)	< 0.001	67,044 (35.7%)	3376 (24.9%)	< 0.001
Junior high school	79,851 (18.1%)	32,617 (16.2%)		30,505 (16.3%)	2112 (15.6%)	
Primary school or lower	191,904 (43.5%)	98,072 (48.8%)		89,999 (48.0%)	8073 (59.5%)	
Marital status						
Unmarried	68,421 (15.5%)	30,423 (15.1%)	< 0.001	28,417 (15.2%)	2006 (14.8%)	< 0.001
Married	329,056 (74.6%)	151,013 (75.1%)		141,564 (75.5%)	9449 (69.7%)	
Divorced or widowed	43,833 (9.9%)	19,673 (9.8%)		17,567 (9.4%)	2106 (15.5%)	
Employment status						
Jobless	128,695 (29.2%)	57,798 (28.7%)	< 0.001	52,780 (28.1%)	5018 (37.0%)	< 0.001
Student	12,936 (2.9%)	5840 (2.9%)		5404 (2.9%)	436 (3.2%)	
Employed or retired	56,378 (12.8%)	21,946 (10.9%)		21,165 (11.3%)	781 (5.8%)	
Self-employed	66,687 (15.1%)	24,635 (12.2%)		23,398 (12.5%)	1,237 (9.1%)	
Informal worker	149,199 (33.8%)	75,227 (37.4%)		70,158 (37.4%)	5,069 (37.4%)	
Other	27,415 (6.2%)	15,663 (7.8%)		14,643 (7.8%)	1,020 (7.5%)	
Monthly household expenditure by quintile						
1st quintile	72,460 (16.4%)	41,935 (20.9%)	< 0.001	38,480 (20.5%)	3455 (25.5%)	< 0.001
2nd quintile	80,993 (18.4%)	40,228 (20.0%)		37,243 (19.9%)	2985 (22.0%)	
3rd quintile	87,415 (19.8%)	39,936 (19.9%)		37,237 (19.9%)	2699 (19.9%)	
4th quintile	94,449 (21.4%)	40,326 (20.1%)		37,866 (20.2%)	2460 (18.1%)	
5th quintile	105,993 (24.0%)	38,684 (19.2%)		36,722 (19.6%)	1962 (14.5%)	
Have difficulty accessing healthcare						
No	361,986 (89.9%)	150,424 (85.9%)	< 0.001	141,514 (86.4%)	8910 (79.2%)	< 0.001
Yes	40,706 (10.1%)	24,654 (14.1%)		22,320 (13.6%)	2334 (20.8%)	
Smoking status						
Every day	118,580 (26.9%)	54,712 (27.2%)	< 0.001	51,538 (27.5%)	3,174 (23.4%)	< 0.001
Not every day	20,187 (4.6%)	9706 (4.8%)		9031 (4.8%)	675 (5.0%)	
Former smoker	24,107 (5.5%)	9630 (4.8%)		8831 (4.7%)	799 (5.9%)	
Never smoked	278,436 (63.1%)	127,061 (63.2%)		118,148 (63.0%)	8913 (65.7%)	
Alcohol use						
Under standard	11,145 (2.5%)	6685 (3.3%)	< 0.001	6121 (3.3%)	564 (4.2%)	< 0.001
More than standard	6261 (1.4%)	6420 (3.2%)		5873 (3.1%)	547 (4.0%)	
No alcohol	423,904 (96.1%)	188,004 (93.5%)		175,554 (93.6%)	12,450 (91.8%)	
Physical activity						
Less active	43,399 (9.8%)	20,216 (10.1%)	0.007	18,743 (10.0%)	1,473 (10.9%)	0.001
Active	397,911 (90.2%)	180,893 (89.9%)		168,805 (90.0%)	12,088 (89.1%)	
Have you ever been diagnosed with lung tuberculosis?						
No	439,019 (99.5%)	200,118 (99.5%)	0.169	186,715 (99.6%)	13,403 (98.8%)	< 0.001
Yes	2,291 (0.5%)	991 (0.5%)		833 (0.4%)	158 (1.2%)	
Have you ever been diagnosed with hypertension?						
No	401,688 (91.0%)	183,702 (91.3%)	< 0.001	172,063 (91.7%)	11,639 (85.8%)	< 0.001
Yes	39,622 (9.0%)	17,407 (8.7%)		15,485 (8.3%)	1922 (14.2%)	
Have you ever been diagnosed with stroke?						
No	435,842 (98.8%)	198,781 (98.8%)	0.006	185,669 (99.0%)	13,112 (96.7%)	< 0.001
Yes	5468 (1.2%)	2328 (1.2%)		1879 (1.0%)	449 (3.3%)	
Have you ever been diagnosed with diabetes mellitus?						
No	430,876 (97.6%)	197,059 (98.0%)	< 0.001	184,027 (98.1%)	13,032 (96.1%)	< 0.001
Yes	10,434 (2.4%)	4050 (2.0%)		3521 (1.9%)	529 (3.9%)	
Have you ever been diagnosed with heart disease?						
No	432,384 (98.0%)	197,557 (98.2%)	< 0.001	184,484 (98.4%)	13,073 (96.4%)	< 0.001
Yes	8926 (2.0%)	3552 (1.8%)		3064 (1.6%)	488 (3.6%)	
Have you ever been diagnosed with asthma?						
No	429,663 (97.4%)	195,512 (97.2%)	< 0.001	182,765 (97.4%)	12,747 (94.0%)	< 0.001
Yes	11,647 (2.6%)	5597 (2.8%)		4783 (2.6%)	814 (6.0%)	
Have you ever been diagnosed with rheumatoid arthritis?						
No	402,771 (91.3%)	184,910 (91.9%)	< 0.001	173,339 (92.4%)	11,571 (85.3%)	< 0.001
Yes	38,539 (8.7%)	16,199 (8.1%)		14,209 (7.6%)	1990 (14.7%)	
Have you ever been diagnosed with cancer?						
No	440,088 (99.7%)	200,586 (99.7%)	0.229	187,113 (99.8%)	13,473 (99.4%)	< 0.001
Yes	1222 (0.3%)	523 (0.3%)		435 (0.2%)	88 (0.6%)	
Have you ever been diagnosed with kidney failure?						
No	439,435 (99.6%)	200,292 (99.6%)	0.284	186,860 (99.6%)	13,432 (99.0%)	< 0.001
Yes	1875 (0.4%)	817 (0.4%)		688 (0.4%)	129 (1.0%)	
Family member with psychosis						
No	437,596 (99.2%)	199,287 (99.1%)	0.010	185,991 (99.2%)	13,296 (98.0%)	< 0.001
Yes	3714 (0.8%)	1822 (0.9%)		1557 (0.8%)	265 (2.0%)	

As the bivariate analysis, the continuous variables between different sex and rurality were compared using t-test, while categorical variables were compared using chi-square tests.

**Table 2:** Multivariable logistic regression results showing the associations between living in areas with coastal hazards and depression. *Source*: Riskesdas 2018 and Podes 2018.

	Model 1^‡^	Model 2^‡^	Model 3^‡^	Model 4^‡^
Living in district with coastal hazards	1.13 [1.10, 1.16]*			
Living in district with coastal abrasion		1.16 [1.13, 1.19]*		
Living in district area with hurricanes			1.14 [1.11, 1.16]*	
Living in district with tidal flooding				1.24 [1.20, 1.28]*
Age group (Ref.: 18–24 years old )				
25–34 years old	0.88 [0.83, 0.92]*	0.88 [0.84, 0.92]*	0.88 [0.84, 0.92]	0.88 [0.84;0.92]
35–44 years old	0.92 [0.87, 0.96]*	0.92 [0.87, 0.96]*	0.92 [0.87, 0.96]*	0.92 [0.87;0.96]*
45–54 years old	0.90 [0.86, 0.95]*	0.90 [0.86, 0.95]*	0.90 [0.86, 0.95]*	0.90 [0.86;0.95]*
55–64 years old	0.74 [0.70, 0.78]*	0.74 [0.70, 0.78]*	0.74 [0.70, 0.78]*	0.74 [0.70;0.78]*
65–74 years old	0.71 [0.67, 0.76]*	0.72 [0.67, 0.76]*	0.71 [0.67, 0.76]*	0.72 [0.67;0.76]*
75 years old or above	0.68 [0.63, 0.73]*	0.68 [0.63, 0.74]*	0.68 [0.63, 0.73]*	0.68 [0.63;0.74]*
Female	2.17 [2.08, 2.25]*	2.16 [2.08, 2.25]*	2.16 [2.08, 2.25]*	2.16 [2.08;2.25]*
Education (Ref.: senior high school or higher)				
Junior high school	1.19 [1.15, 1.23]*	1.19 [1.15, 1.23]*	1.19 [1.15, 1.23]*	1.19 [1.15;1.23]*
Primary school or lower	1.38 [1.34, 1.43]*	1.38 [1.34, 1.43]*	1.38 [1.34, 1.43]*	1.38 [1.34;1.43]*
Marital status (Ref.: unmarried)				
Married	0.78 [0.75, 0.81]*	0.78 [0.75, 0.81]*	0.78 [0.75, 0.81]*	0.78 [0.75;0.81]*
Divorced or widowed	1.13 [1.07, 1.19]*	1.13 [1.06, 1.19]*	1.13 [1.07, 1.19]*	1.12 [1.06;1.19]*
Employment status (Ref.: jobless)				
Student	0.99 [0.92, 1.07]	1.00 [0.93, 1.07]	0.99 [0.92, 1.07]	0.99 [0.92;1.07]
Employed or retired	0.66 [0.63, 0.70]*	0.67 [0.64, 0.70]*	0.66 [0.63, 0.70]*	0.67 [0.63;0.70]*
Self-employed	0.82 [0.78, 0.85]*	0.82 [0.78, 0.85]*	0.82 [0.78, 0.85]*	0.82 [0.78;0.85]*
Informal worker	0.84 [0.82, 0.87]*	0.84 [0.82, 0.87]*	0.84 [0.82, 0.87]*	0.84 [0.82;0.87]*
Other	0.87 [0.83, 0.92]*	0.87 [0.83, 0.91]*	0.87 [0.83, 0.92]*	0.87 [0.83;0.92]*
Monthly household expenditure (Ref.: 1st quintile)				
2nd quintile	0.98 [0.95, 1.02]	0.98 [0.95, 1.02]	0.98 [0.95, 1.02]	0.98 [0.95;1.02]
3rd quintile	0.96 [0.93, 1.00]	0.96 [0.93, 1.00]	0.97 [0.93, 1.00]	0.97 [0.93, 1.00]
4th quintile	0.89 [0.86, 0.92]*	0.89 [0.86, 0.92]*	0.89 [0.86, 0.92]*	0.89 [0.86, 0.92]*
5th quintile	0.78 [0.75, 0.81]*	0.79 [0.76, 0.82]*	0.79 [0.76, 0.82]*	0.79 [0.76, 0.82]*
Smoking status (Ref.: every day)				
Not every day	1.00 [0.94, 1.06]	1.00 [0.94, 1.06]	1.00 [0.94, 1.06]	1.00 [0.94, 1.06]
Former smoker	1.24 [1.18, 1.31]*	1.25 [1.18, 1.31]*	1.24 [1.18, 1.31]*	1.25 [1.19, 1.32]*
Never smoked	0.62 [0.60, 0.65]*	0.62 [0.60, 0.65]*	0.62 [0.60, 0.65]*	0.62 [0.60, 0.65]*
Alcohol use (Ref.: under standard)				
More than standard	0.97 [0.89, 1.06]	0.97 [0.89, 1.06]	0.97 [0.89, 1.06]	0.97 [0.89, 1.06]
No alcohol	0.55 [0.52, 0.59]*	0.55 [0.52, 0.58]*	0.55 [0.52, 0.59]*	0.55 [0.51, 0.58]*
Physically active	0.90 [0.87, 0.94]*	0.91 [0.87, 0.94]*	0.90 [0.87, 0.94]*	0.90 [0.87, 0.94]*
Ever diagnosed with lung tuberculosis	2.20 [1.97, 2.45]*	2.20 [1.97, 2.45]*	2.20 [1.97, 2.45]*	2.20 [1.97, 2.45]*
Ever diagnosed with hypertension	1.42 [1.37, 1.47]*	1.42 [1.37, 1.47]*	1.42 [1.37, 1.47]*	1.42 [1.37, 1.47]*
Ever diagnosed with stroke	2.45 [2.29, 2.62]*	2.45 [2.29, 2.62]*	2.45 [2.29, 2.62]*	2.45 [2.29, 2.62]*
Ever diagnosed with diabetes	1.64 [1.54, 1.73]*	1.64 [1.54, 1.73]*	1.64 [1.54, 1.73]*	1.64 [1.54, 1.73]*
Ever diagnosed with heart disease	1.57 [1.48, 1.67]*	1.57 [1.48, 1.67]*	1.57 [1.48, 1.67]*	1.58 [1.48, 1.67]*
Ever diagnosed with asthma	2.02 [1.92, 2.13]*	2.02 [1.92, 2.13]*	2.02 [1.92, 2.13]*	2.03 [1.92, 2.13]*
Ever diagnosed with rheumatoid arthritis	1.85 [1.79, 1.91]*	1.85 [1.79, 1.91]*	1.85 [1.79, 1.91]*	1.85 [1.79, 1.91]*
Ever diagnosed with cancer	2.33 [2.02, 2.69]*	2.33 [2.02, 2.69]*	2.34 [2.02, 2.69]*	2.34 [2.02, 2.69]*
Ever diagnosed with kidney failure	2.08 [1.85, 2.34]*	2.08 [1.85, 2.34]*	2.08 [1.85, 2.34]*	2.08 [1.85, 2.34]*
Family member with psychosis	2.33 [2.14, 2.53]*	2.33 [2.14, 2.53]*	2.33 [2.14, 2.54]*	2.34 [2.14, 2.54]*
Have difficulty accessing healthcare	1.37 [1.33, 1.42]*	1.37 [1.33, 1.42]*	1.37 [1.33, 1.41]*	1.37 [1.33, 1.41]*
Intercept	0.11 [0.10, 0.12]*	0.11 [0.10, 0.12]*	0.11 [0.10, 0.12]*	0.11 [0.10, 0.12]*
Number of observations	577,770	577,770	577,770	577,770

^‡^Presented are Odds ratio and 95% Confidence intervals. ^*^p < 0.01 (Bonferroni correction)

**Table 3:** Multivariable logistics regression results showing factors associated with depression in areas with (1) coastal hazards, (2) coastal abrasions, (3) hurricanes, and (4) tidal flooding. *Source*: Riskesdas 2018 and Podes 2018.

	Individuals living in districts with coastal hazards	Individuals living in districts with coastal abrasion	Individuals living in coastal districts with hurricane	Individuals living in areas with coastal districts with tidal flooding
Age group (Ref.: 18–24)				
25–34	0.88 [0.81;0.95]*	0.86 [0.79;0.94]*	0.87 [0.80;0.95]*	0.91 [0.82;1.02]
35–44	0.94 [0.87;1.03]	0.93 [0.85;1.02]	0.94 [0.86;1.03]	0.98 [0.87;1.10]
45–54	0.95 [0.87;1.04]	0.95 [0.87;1.05]	0.95 [0.87;1.04]	1.00 [0.89;1.13]
55–64	0.76 [0.69;0.84]*	0.76 [0.69;0.85]*	0.77 [0.69;0.85]*	0.81 [0.71;0.93]*
65–74	0.73 [0.65;0.82]*	0.73 [0.65;0.83]*	0.74 [0.66;0.83]*	0.83 [0.72;0.97]
75+	0.72 [0.63;0.82]*	0.73 [0.63;0.85]*	0.72 [0.62;0.83]*	0.81 [0.67;0.97]*
Female	2.03 [1.90;2.18]*	1.96 [1.81;2.11]*	2.05 [1.90;2.20]*	1.97 [1.79;2.16]*
Education (Ref.: senior high school or higher)				
Junior high school	1.20 [1.12;1.28]*	1.22 [1.14;1.31]*	1.23 [1.15;1.31]*	1.29 [1.18;1.41]*
Primary school or lower	1.39 [1.32;1.47]*	1.42 [1.34;1.51]*	1.41 [1.33;1.49]*	1.43 [1.33;1.55]*
Marital status (Ref.: unmarried)				
Married	0.80 [0.74;0.86]*	0.79 [0.73;0.86]*	0.81 [0.75;0.87]*	0.81 [0.73;0.90]*
Divorced or widowed	1.14 [1.04;1.26]*	1.10 [0.99;1.23]	1.12 [1.02;1.24]	1.16 [1.02;1.32]
Employment status (Ref.: jobless)				
Student	1.06 [0.94;1.19]	1.01 [0.87;1.16]	1.03 [0.91;1.18]	1.10 [0.93;1.31]
Employed or retired	0.65 [0.59;0.71]*	0.67 [0.60;0.74]*	0.65 [0.59;0.71]*	0.71 [0.62;0.80]*
Self-employed	0.77 [0.72;0.83]*	0.81 [0.74;0.87]*	0.79 [0.73;0.86]*	0.86 [0.78;0.95]*
Informal worker	0.85 [0.80;0.89]*	0.81 [0.77;0.86]*	0.86 [0.81;0.90]*	0.85 [0.79;0.91]*
Other	0.87 [0.80;0.94]*	0.85 [0.78;0.93]*	0.89 [0.82;0.96]*	0.93 [0.84;1.03]
Monthly household expenditure (Ref.: 1st quintile)				
2nd quintile	0.93 [0.88;0.99]	0.96 [0.90;1.02]	0.93 [0.88;0.99]	0.95 [0.88;1.03]
3rd quintile	0.90 [0.85;0.95]*	0.92 [0.86;0.98]	0.90 [0.85;0.96]*	0.93 [0.86;1.01]
4th quintile	0.82 [0.77;0.87]*	0.84 [0.78;0.90]*	0.84 [0.79;0.89]*	0.86 [0.79;0.94]*
5th quintile	0.76 [0.71;0.81]*	0.79 [0.74;0.86]*	0.77 [0.72;0.83]*	0.81 [0.74;0.89]*
Smoking status (Ref: every day)				
Not every day	1.10 [1.00;1.22]	1.10 [0.99;1.23]	1.08 [0.98;1.20]	1.11 [0.98;1.27]
Former smoker	1.23 [1.12;1.35]*	1.22 [1.10;1.36]*	1.24 [1.13;1.37]*	1.15 [1.00;1.31]
Never smoked	0.66 [0.61;0.71]*	0.68 [0.63;0.74]*	0.65 [0.61;0.71]*	0.66 [0.60;0.73]*
Alcohol use (Ref.: under standard)				
More than standard	0.99 [0.86;1.13]	0.95 [0.81;1.10]	0.99 [0.86;1.13]	0.90 [0.75;1.09]
No alcohol	0.59 [0.54;0.66]*	0.59 [0.53;0.66]*	0.58 [0.52;0.64]*	0.57 [0.50;0.66]*
Physically active	0.90 [0.85;0.96]*	0.90 [0.84;0.96]*	0.90 [0.84;0.96]*	0.93 [0.85;1.02]
Ever diagnosed with lung tuberculosis	2.11 [1.73;2.56]*	2.22 [1.79;2.74]*	2.05 [1.66;2.52]*	2.22 [1.71;2.89]*
Ever diagnosed with hypertension	1.35 [1.27;1.44]*	1.36 [1.27;1.46]*	1.36 [1.28;1.46]*	1.36 [1.25;1.49]*
Ever diagnosed with stroke	2.50 [2.22;2.82]*	2.41 [2.10;2.76]*	2.41 [2.12;2.74]*	2.35 [1.97;2.80]*
Ever diagnosed with diabetes	1.67 [1.51;1.86]*	1.77 [1.57;1.98]*	1.69 [1.51;1.89]*	1.84 [1.59;2.12]*
Ever diagnosed with heart disease	1.66 [1.49;1.85]*	1.62 [1.43;1.83]*	1.65 [1.46;1.85]*	1.48 [1.26;1.74]*
Ever diagnosed with asthma	2.06 [1.89;2.25]*	2.10 [1.91;2.31]*	2.10 [1.92;2.30]*	2.22 [1.97;2.50]*
Ever diagnosed with rheumatoid arthritis	1.78 [1.68;1.89]*	1.80 [1.69;1.92]*	1.75 [1.65;1.87]*	1.71 [1.57;1.85]*
Ever diagnosed with cancer	2.40 [1.88;3.08]*	2.64 [2.02;3.46]*	2.28 [1.73;2.99]*	2.62 [1.84;3.71]*
Ever diagnosed with kidney failure	1.87 [1.51;2.31]*	2.11 [1.68;2.65]*	1.91 [1.52;2.38]*	2.03 [1.54;2.69]*
Family member with psychosis	2.14 [1.85;2.49]*	2.13 [1.81;2.51]*	2.18 [1.86;2.56]*	2.10 [1.69;2.61]*
Difficulty to access healthcare	1.45 [1.38;1.53]*	1.41 [1.33;1.49]*	1.45 [1.38;1.53]*	1.44 [1.35;1.54]*
Intercept	0.11 [0.10;0.13]*	0.12 [0.10;0.13]*	0.11 [0.09;0.13]*	0.11 [0.09;0.13]*
Number of observations	175,078	138,286	155,488	84,498

^‡^Presented are Odds ratio and 95% Confidence intervals. ^*^p < 0.01 (Bonferroni correction)

## Electronic supplementary material

Below is the link to the electronic supplementary material.

Supplementary Material 1

## Data Availability

Indonesia Basic Health Survey (Riset Kesehatan Dasar or Riskesdas) data are available through the Health Policy and Development Agency, Ministry of Health, Republic of Indonesia, at https://layanandata.kemkes.go.id/. The form for data requests can be found in https://layanandata.kemkes.go.id/request.
