# Supplementary material for: Association between climate related hazards and depression among coastal communities in Indonesia
Source: Sci Rep. 2025 Feb 27;15:6998. doi: 10.1038/s41598-025-89298-1 (PMC11868409; doi:10.1038/s41598-025-89298-1)

**Supplementary Table S1** Characteristics of respondents by areas of living

|  | **Abrasion** | | | **Hurricane** | | | **Tidal flooding** | | |
| --- | --- | --- | --- | --- | --- | --- | --- | --- | --- |
|  | Coastal area without abrasion | Coastal area with abrasion | Test | Coastal area without hurricane | Coastal area with hurricane | Test | Coastal area without tidal flooding | Coastal area with tidal flooding | Test |
| N (%) | 483,162 (75.2%) | 159,257 (24.8%) |  | 462,886 (72.1%) | 179,533 (27.9%) |  | 543,913 (84.7%) | 98,506 (15.3%) |  |
| Depression |  |  |  |  |  |  |  |  |  |
| No | 455,655 (94.3%) | 148,149 (93.0%) | <0.001 | 436,567 (94.3%) | 167,237 (93.2%) | <0.001 | 512,655 (94.3%) | 91,149 (92.5%) | <0.001 |
| Yes | 27,507 (5.7%) | 11,108 (7.0%) |  | 26,319 (5.7%) | 12,296 (6.8%) |  | 31,258 (5.7%) | 7,357 (7.5%) |  |
| Age |  |  |  |  |  |  |  |  |  |
| 18-24 years old | 65,575 (13.6%) | 21,133 (13.3%) | <0.001 | 62,691 (13.5%) | 24,017 (13.4%) | <0.001 | 73,502 (13.5%) | 13,206 (13.4%) | <0.001 |
| 25-34 years old | 99,029 (20.5%) | 33,397 (21.0%) |  | 95,279 (20.6%) | 37,147 (20.7%) |  | 111,880 (20.6%) | 20,546 (20.9%) |  |
| 35-44 years old | 114,232 (23.6%) | 37,462 (23.5%) |  | 109,526 (23.7%) | 42,168 (23.5%) |  | 128,622 (23.6%) | 23,072 (23.4%) |  |
| 45-54 years old | 96,673 (20.0%) | 31,264 (19.6%) |  | 92,534 (20.0%) | 35,403 (19.7%) |  | 108,648 (20.0%) | 19,289 (19.6%) |  |
| 55-64 years old | 64,321 (13.3%) | 20,866 (13.1%) |  | 61,651 (13.3%) | 23,536 (13.1%) |  | 72,188 (13.3%) | 12,999 (13.2%) |  |
| 65-74 years old | 29,344 (6.1%) | 10,242 (6.4%) |  | 27,924 (6.0%) | 11,662 (6.5%) |  | 33,205 (6.1%) | 6,381 (6.5%) |  |
| 75 years old and above | 13,988 (2.9%) | 4,893 (3.1%) |  | 13,281 (2.9%) | 5,600 (3.1%) |  | 15,868 (2.9%) | 3,013 (3.1%) |  |
| Gender |  |  |  |  |  |  |  |  |  |
| Male | 229,657 (47.5%) | 75,044 (47.1%) | 0.004 | 220,216 (47.6%) | 84,485 (47.1%) | <0.001 | 258,298 (47.5%) | 46,403 (47.1%) | 0.027 |
| Female | 253,505 (52.5%) | 84,213 (52.9%) |  | 242,670 (52.4%) | 95,048 (52.9%) |  | 285,615 (52.5%) | 52,103 (52.9%) |  |
| Education |  |  |  |  |  |  |  |  |  |
| Senior high school or higher | 186,809 (38.7%) | 53,166 (33.4%) | <0.001 | 178,684 (38.6%) | 61,291 (34.1%) | <0.001 | 208,226 (38.3%) | 31,749 (32.2%) | <0.001 |
| Junior high school | 86,617 (17.9%) | 25,851 (16.2%) |  | 83,588 (18.1%) | 28,880 (16.1%) |  | 96,320 (17.7%) | 16,148 (16.4%) |  |
| Primary school or lower | 209,736 (43.4%) | 80,240 (50.4%) |  | 200,614 (43.3%) | 89,362 (49.8%) |  | 239,367 (44.0%) | 50,609 (51.4%) |  |
| Marital status |  |  |  |  |  |  |  |  |  |
| Unmarried | 75,522 (15.6%) | 23,322 (14.6%) | <0.001 | 71,923 (15.5%) | 26,921 (15.0%) | <0.001 | 84,433 (15.5%) | 14,411 (14.6%) | <0.001 |
| Married | 359,807 (74.5%) | 120,262 (75.5%) |  | 345,158 (74.6%) | 134,911 (75.1%) |  | 405,792 (74.6%) | 74,277 (75.4%) |  |
| Divorced or widowed | 47,833 (9.9%) | 15,673 (9.8%) |  | 45,805 (9.9%) | 17,701 (9.9%) |  | 53,688 (9.9%) | 9,818 (10.0%) |  |
| Employment status |  |  |  |  |  |  |  |  |  |
| Jobless | 139,890 (29.0%) | 46,603 (29.3%) | <0.001 | 135,395 (29.3%) | 51,098 (28.5%) | <0.001 | 158,424 (29.1%) | 28,069 (28.5%) | <0.001 |
| Student | 14,421 (3.0%) | 4,355 (2.7%) |  | 13,580 (2.9%) | 5,196 (2.9%) |  | 15,982 (2.9%) | 2,794 (2.8%) |  |
| Employed or retired | 62,768 (13.0%) | 15,556 (9.8%) |  | 59,199 (12.8%) | 19,125 (10.7%) |  | 69,076 (12.7%) | 9,248 (9.4%) |  |
| Self-employed | 73,023 (15.1%) | 18,299 (11.5%) |  | 69,793 (15.1%) | 21,529 (12.0%) |  | 79,726 (14.7%) | 11,596 (11.8%) |  |
| Informal worker | 162,793 (33.7%) | 61,633 (38.7%) |  | 155,498 (33.6%) | 68,928 (38.4%) |  | 184,978 (34.0%) | 39,448 (40.0%) |  |
| Other | 30,267 (6.3%) | 12,811 (8.0%) |  | 29,421 (6.4%) | 13,657 (7.6%) |  | 35,727 (6.6%) | 7,351 (7.5%) |  |
| Monthly household expenditure by quintile |  |  |  |  |  |  |  |  |  |
| 1st quintile | 79,670 (16.5%) | 34,725 (21.8%) | <0.001 | 74,939 (16.2%) | 39,456 (22.0%) | <0.001 | 91,752 (16.9%) | 22,643 (23.0%) | <0.001 |
| 2nd quintile | 88,577 (18.3%) | 32,644 (20.5%) |  | 84,327 (18.2%) | 36,894 (20.5%) |  | 100,268 (18.4%) | 20,953 (21.3%) |  |
| 3rd quintile | 95,169 (19.7%) | 32,182 (20.2%) |  | 91,707 (19.8%) | 35,644 (19.9%) |  | 107,527 (19.8%) | 19,824 (20.1%) |  |
| 4th quintile | 102,617 (21.2%) | 32,158 (20.2%) |  | 99,675 (21.5%) | 35,100 (19.6%) |  | 115,976 (21.3%) | 18,799 (19.1%) |  |
| 5th quintile | 117,129 (24.2%) | 27,548 (17.3%) |  | 112,238 (24.2%) | 32,439 (18.1%) |  | 128,390 (23.6%) | 16,287 (16.5%) |  |
| Smoking status |  |  |  |  |  |  |  |  |  |
| Every day | 128,774 (26.7%) | 44,518 (28.0%) | <0.001 | 124,376 (26.9%) | 48,916 (27.2%) | <0.001 | 145,243 (26.7%) | 28,049 (28.5%) | <0.001 |
| Not every day | 22,195 (4.6%) | 7,698 (4.8%) |  | 21,242 (4.6%) | 8,651 (4.8%) |  | 25,099 (4.6%) | 4,794 (4.9%) |  |
| Former smoker | 26,331 (5.4%) | 7,406 (4.7%) |  | 25,198 (5.4%) | 8,539 (4.8%) |  | 29,471 (5.4%) | 4,266 (4.3%) |  |
| Never smoked | 305,862 (63.3%) | 99,635 (62.6%) |  | 292,070 (63.1%) | 113,427 (63.2%) |  | 344,100 (63.3%) | 61,397 (62.3%) |  |
| Alcohol use |  |  |  |  |  |  |  |  |  |
| Under standard | 12,826 (2.7%) | 5,004 (3.1%) | <0.001 | 11,724 (2.5%) | 6,106 (3.4%) | <0.001 | 14,748 (2.7%) | 3,082 (3.1%) | <0.001 |
| More than standard | 7,645 (1.6%) | 5,036 (3.2%) |  | 6,937 (1.5%) | 5,744 (3.2%) |  | 9,697 (1.8%) | 2,984 (3.0%) |  |
| No alcohol | 462,691 (95.8%) | 149,217 (93.7%) |  | 444,225 (96.0%) | 167,683 (93.4%) |  | 519,468 (95.5%) | 92,440 (93.8%) |  |
| Physical activity |  |  |  |  |  |  |  |  |  |
| Less active | 47,285 (9.8%) | 16,330 (10.3%) | <0.001 | 46,011 (9.9%) | 17,604 (9.8%) | 0.105 | 54,392 (10.0%) | 9,223 (9.4%) | <0.001 |
| Active | 435,877 (90.2%) | 142,927 (89.7%) |  | 416,875 (90.1%) | 161,929 (90.2%) |  | 489,521 (90.0%) | 89,283 (90.6%) |  |
| Have you ever been diagnosed with lung tuberculosis? |  |  |  |  |  |  |  |  |  |
| No | 480,687 (99.5%) | 158,450 (99.5%) | 0.789 | 460,496 (99.5%) | 178,641 (99.5%) | 0.326 | 541,134 (99.5%) | 98,003 (99.5%) | 0.990 |
| Yes | 2,475 (0.5%) | 807 (0.5%) |  | 2,390 (0.5%) | 892 (0.5%) |  | 2,779 (0.5%) | 503 (0.5%) |  |
| Have you ever been diagnosed with hypertension? |  |  |  |  |  |  |  |  |  |
| No | 439,692 (91.0%) | 145,698 (91.5%) | <0.001 | 421,064 (91.0%) | 164,326 (91.5%) | <0.001 | 495,271 (91.1%) | 90,119 (91.5%) | <0.001 |
| Yes | 43,470 (9.0%) | 13,559 (8.5%) |  | 41,822 (9.0%) | 15,207 (8.5%) |  | 48,642 (8.9%) | 8,387 (8.5%) |  |
| Have you ever been diagnosed with stroke? |  |  |  |  |  |  |  |  |  |
| No | 477,145 (98.8%) | 157,478 (98.9%) | <0.001 | 457,140 (98.8%) | 177,483 (98.9%) | 0.001 | 537,191 (98.8%) | 97,432 (98.9%) | <0.001 |
| Yes | 6,017 (1.2%) | 1,779 (1.1%) |  | 5,746 (1.2%) | 2,050 (1.1%) |  | 6,722 (1.2%) | 1,074 (1.1%) |  |
| Have you ever been diagnosed with diabetes mellitus? |  |  |  |  |  |  |  |  |  |
| No | 471,801 (97.6%) | 156,134 (98.0%) | <0.001 | 451,896 (97.6%) | 176,039 (98.1%) | <0.001 | 531,275 (97.7%) | 96,660 (98.1%) | <0.001 |
| Yes | 11,361 (2.4%) | 3,123 (2.0%) |  | 10,990 (2.4%) | 3,494 (1.9%) |  | 12,638 (2.3%) | 1,846 (1.9%) |  |
| Have you ever been diagnosed with heart disease? |  |  |  |  |  |  |  |  |  |
| No | 473,472 (98.0%) | 156,469 (98.2%) | <0.001 | 453,537 (98.0%) | 176,404 (98.3%) | <0.001 | 533,070 (98.0%) | 96,871 (98.3%) | <0.001 |
| Yes | 9,690 (2.0%) | 2,788 (1.8%) |  | 9,349 (2.0%) | 3,129 (1.7%) |  | 10,843 (2.0%) | 1,635 (1.7%) |  |
| Have you ever been diagnosed with asthma? |  |  |  |  |  |  |  |  |  |
| No | 470,305 (97.3%) | 154,870 (97.2%) | 0.045 | 450,605 (97.3%) | 174,570 (97.2%) | 0.013 | 529,315 (97.3%) | 95,860 (97.3%) | 0.968 |
| Yes | 12,857 (2.7%) | 4,387 (2.8%) |  | 12,281 (2.7%) | 4,963 (2.8%) |  | 14,598 (2.7%) | 2,646 (2.7%) |  |
| Have you ever been diagnosed with rheumatoid arthritis? |  |  |  |  |  |  |  |  |  |
| No | 441,078 (91.3%) | 146,603 (92.1%) | <0.001 | 422,261 (91.2%) | 165,420 (92.1%) | <0.001 | 496,888 (91.4%) | 90,793 (92.2%) | <0.001 |
| Yes | 42,084 (8.7%) | 12,654 (7.9%) |  | 40,625 (8.8%) | 14,113 (7.9%) |  | 47,025 (8.6%) | 7,713 (7.8%) |  |
| Have you ever been diagnosed with cancer? |  |  |  |  |  |  |  |  |  |
| No | 481,822 (99.7%) | 158,852 (99.7%) | 0.126 | 461,593 (99.7%) | 179,081 (99.7%) | 0.057 | 542,402 (99.7%) | 98,272 (99.8%) | 0.026 |
| Yes | 1,340 (0.3%) | 405 (0.3%) |  | 1,293 (0.3%) | 452 (0.3%) |  | 1,511 (0.3%) | 234 (0.2%) |  |
| Have you ever been diagnosed with kidney failure? |  |  |  |  |  |  |  |  |  |
| No | 481,111 (99.6%) | 158,616 (99.6%) | 0.239 | 460,925 (99.6%) | 178,802 (99.6%) | 0.359 | 541,643 (99.6%) | 98,084 (99.6%) | 0.621 |
| Yes | 2,051 (0.4%) | 641 (0.4%) |  | 1,961 (0.4%) | 731 (0.4%) |  | 2,270 (0.4%) | 422 (0.4%) |  |
| Family member with psychosis |  |  |  |  |  |  |  |  |  |
| No | 479,080 (99.2%) | 157,803 (99.1%) | 0.011 | 458,910 (99.1%) | 177,973 (99.1%) | 0.698 | 539,195 (99.1%) | 97,688 (99.2%) | 0.248 |
| Yes | 4,082 (0.8%) | 1,454 (0.9%) |  | 3,976 (0.9%) | 1,560 (0.9%) |  | 4,718 (0.9%) | 818 (0.8%) |  |
| Have difficulty accessing healthcare |  |  |  |  |  |  |  |  |  |
| No | 394,388 (89.7%) | 118,022 (85.3%) | <0.001 | 379,894 (90.0%) | 132,516 (85.2%) | <0.001 | 441,267 (89.5%) | 71,143 (84.2%) | <0.001 |
| Yes | 45,096 (10.3%) | 20,264 (14.7%) |  | 42,388 (10.0%) | 22,972 (14.8%) |  | 52,005 (10.5%) | 13,355 (15.8%) |  |

**Supplementary Table S2** Characteristics of respondents living in (1) coastal areas with abrasion, (2) coastal areas with hurricane and (3) coastal areas with tidal flooding by the presence of depression

| **Variables** | **Respondents living in coastal areas with abrasion** | | | **Respondents living in coastal areas with hurricane** | | | **Respondents living in coastal areas with tidal flooding** | | |
| --- | --- | --- | --- | --- | --- | --- | --- | --- | --- |
|  | Not depressed | Depressed | Test | Not depressed | Depressed | Test | Not depressed | Depressed | Test |
| N (%) | 148,149 (93.0%) | 11,108 (7.0%) |  | 167,237 (93.2%) | 12,296 (6.8%) |  | 91,149 (92.5%) | 7,357 (7.5%) |  |
| Age |  |  |  |  |  |  |  |  |  |
| 18-24 years old | 19,640 (13.3%) | 1,493 (13.4%) | <0.001 | 22,374 (13.4%) | 1,643 (13.4%) | <0.001 | 12,252 (13.4%) | 954 (13.0%) | <0.001 |
| 25-34 years old | 31,479 (21.2%) | 1,918 (17.3%) |  | 35,043 (21.0%) | 2,104 (17.1%) |  | 19,268 (21.1%) | 1,278 (17.4%) |  |
| 35-44 years old | 35,021 (23.6%) | 2,441 (22.0%) |  | 39,429 (23.6%) | 2,739 (22.3%) |  | 21,429 (23.5%) | 1,643 (22.3%) |  |
| 45-54 years old | 28,981 (19.6%) | 2,283 (20.6%) |  | 32,883 (19.7%) | 2,520 (20.5%) |  | 17,783 (19.5%) | 1,506 (20.5%) |  |
| 55-64 years old | 19,285 (13.0%) | 1,581 (14.2%) |  | 21,789 (13.0%) | 1,747 (14.2%) |  | 11,969 (13.1%) | 1,030 (14.0%) |  |
| 65-74 years old | 9,347 (6.3%) | 895 (8.1%) |  | 10,662 (6.4%) | 1,000 (8.1%) |  | 5,765 (6.3%) | 616 (8.4%) |  |
| 75 years old and above | 4,396 (3.0%) | 497 (4.5%) |  | 5,057 (3.0%) | 543 (4.4%) |  | 2,683 (2.9%) | 330 (4.5%) |  |
| Gender |  |  |  |  |  |  |  |  |  |
| Male | 70,915 (47.9%) | 4,129 (37.2%) | <0.001 | 79,883 (47.8%) | 4,602 (37.4%) | <0.001 | 43,637 (47.9%) | 2,766 (37.6%) | <0.001 |
| Female | 77,234 (52.1%) | 6,979 (62.8%) |  | 87,354 (52.2%) | 7,694 (62.6%) |  | 47,512 (52.1%) | 4,591 (62.4%) |  |
| Education |  |  |  |  |  |  |  |  |  |
| Senior high school or higher | 50,521 (34.1%) | 2,645 (23.8%) | <0.001 | 58,337 (34.9%) | 2,954 (24.0%) | <0.001 | 30,085 (33.0%) | 1,664 (22.6%) | <0.001 |
| Junior high school | 24,115 (16.3%) | 1,736 (15.6%) |  | 26,965 (16.1%) | 1,915 (15.6%) |  | 14,962 (16.4%) | 1,186 (16.1%) |  |
| Primary school or lower | 73,513 (49.6%) | 6,727 (60.6%) |  | 81,935 (49.0%) | 7,427 (60.4%) |  | 46,102 (50.6%) | 4,507 (61.3%) |  |
| Marital status |  |  |  |  |  |  |  |  |  |
| Unmarried | 21,715 (14.7%) | 1,607 (14.5%) | <0.001 | 25,142 (15.0%) | 1,779 (14.5%) | <0.001 | 13,402 (14.7%) | 1,009 (13.7%) | <0.001 |
| Married | 112,462 (75.9%) | 7,800 (70.2%) |  | 126,279 (75.5%) | 8,632 (70.2%) |  | 69,089 (75.8%) | 5,188 (70.5%) |  |
| Divorced or widowed | 13,972 (9.4%) | 1,701 (15.3%) |  | 15,816 (9.5%) | 1,885 (15.3%) |  | 8,658 (9.5%) | 1,160 (15.8%) |  |
| Employment status |  |  |  |  |  |  |  |  |  |
| Jobless | 42,402 (28.6%) | 4,201 (37.8%) | <0.001 | 46,638 (27.9%) | 4,460 (36.3%) | <0.001 | 25,421 (27.9%) | 2,648 (36.0%) | <0.001 |
| Student | 4,019 (2.7%) | 336 (3.0%) |  | 4,811 (2.9%) | 385 (3.1%) |  | 2,564 (2.8%) | 230 (3.1%) |  |
| Employed or retired | 14,969 (10.1%) | 587 (5.3%) |  | 18,443 (11.0%) | 682 (5.5%) |  | 8,873 (9.7%) | 375 (5.1%) |  |
| Self-employed | 17,298 (11.7%) | 1,001 (9.0%) |  | 20,413 (12.2%) | 1,116 (9.1%) |  | 10,899 (12.0%) | 697 (9.5%) |  |
| Informal worker | 57,505 (38.8%) | 4,128 (37.2%) |  | 64,195 (38.4%) | 4,733 (38.5%) |  | 36,606 (40.2%) | 2,842 (38.6%) |  |
| Other | 11,956 (8.1%) | 855 (7.7%) |  | 12,737 (7.6%) | 920 (7.5%) |  | 6,786 (7.4%) | 565 (7.7%) |  |
| Monthly household expenditure  by quintile |  |  |  |  |  |  |  |  |  |
| 1st quintile | 31,874 (21.5%) | 2,851 (25.7%) | <0.001 | 36,196 (21.6%) | 3,260 (26.5%) | <0.001 | 20,649 (22.7%) | 1,994 (27.1%) | <0.001 |
| 2nd quintile | 30,153 (20.4%) | 2,491 (22.4%) |  | 34,142 (20.4%) | 2,752 (22.4%) |  | 19,294 (21.2%) | 1,659 (22.5%) |  |
| 3rd quintile | 29,943 (20.2%) | 2,239 (20.2%) |  | 33,213 (19.9%) | 2,431 (19.8%) |  | 18,340 (20.1%) | 1,484 (20.2%) |  |
| 4th quintile | 30,119 (20.3%) | 2,039 (18.4%) |  | 32,909 (19.7%) | 2,191 (17.8%) |  | 17,534 (19.2%) | 1,265 (17.2%) |  |
| 5th quintile | 26,060 (17.6%) | 1,488 (13.4%) |  | 30,777 (18.4%) | 1,662 (13.5%) |  | 15,332 (16.8%) | 955 (13.0%) |  |
| Smoking status |  |  |  |  |  |  |  |  |  |
| Every day | 41,883 (28.3%) | 2,635 (23.7%) | <0.001 | 46,008 (27.5%) | 2,908 (23.6%) | <0.001 | 26,237 (28.8%) | 1,812 (24.6%) | <0.001 |
| Not every day | 7,151 (4.8%) | 547 (4.9%) |  | 8,046 (4.8%) | 605 (4.9%) |  | 4,414 (4.8%) | 380 (5.2%) |  |
| Former smoker | 6,770 (4.6%) | 636 (5.7%) |  | 7,819 (4.7%) | 720 (5.9%) |  | 3,871 (4.2%) | 395 (5.4%) |  |
| Never smoked | 92,345 (62.3%) | 7,290 (65.6%) |  | 105,364 (63.0%) | 8,063 (65.6%) |  | 56,627 (62.1%) | 4,770 (64.8%) |  |
| Alcohol use |  |  |  |  |  |  |  |  |  |
| Under standard | 4,565 (3.1%) | 439 (4.0%) | <0.001 | 5,575 (3.3%) | 531 (4.3%) | <0.001 | 2,779 (3.0%) | 303 (4.1%) | <0.001 |
| More than standard | 4,615 (3.1%) | 421 (3.8%) |  | 5,237 (3.1%) | 507 (4.1%) |  | 2,719 (3.0%) | 265 (3.6%) |  |
| No alcohol | 138,969 (93.8%) | 10,248 (92.3%) |  | 156,425 (93.5%) | 11,258 (91.6%) |  | 85,651 (94.0%) | 6,789 (92.3%) |  |
| Physical activity |  |  |  |  |  |  |  |  |  |
| Less active | 15,085 (10.2%) | 1,245 (11.2%) | <0.001 | 16,303 (9.7%) | 1,301 (10.6%) | 0.003 | 8,506 (9.3%) | 717 (9.7%) | 0.241 |
| Active | 133,064 (89.8%) | 9,863 (88.8%) |  | 150,934 (90.3%) | 10,995 (89.4%) |  | 82,643 (90.7%) | 6,640 (90.3%) |  |
| Have you ever been diagnosed with lung tuberculosis? |  |  |  |  |  |  |  |  |  |
| No | 147,478 (99.5%) | 10,972 (98.8%) | <0.001 | 166,487 (99.6%) | 12,154 (98.8%) | <0.001 | 90,736 (99.5%) | 7,267 (98.8%) | <0.001 |
| Yes | 671 (0.5%) | 136 (1.2%) |  | 750 (0.4%) | 142 (1.2%) |  | 413 (0.5%) | 90 (1.2%) |  |
| Have you ever been diagnosed with hypertension? |  |  |  |  |  |  |  |  |  |
| No | 136,157 (91.9%) | 9,541 (85.9%) | <0.001 | 153,729 (91.9%) | 10,597 (86.2%) | <0.001 | 83,792 (91.9%) | 6,327 (86.0%) | <0.001 |
| Yes | 11,992 (8.1%) | 1,567 (14.1%) |  | 13,508 (8.1%) | 1,699 (13.8%) |  | 7,357 (8.1%) | 1,030 (14.0%) |  |
| Have you ever been diagnosed with stroke? |  |  |  |  |  |  |  |  |  |
| No | 146,722 (99.0%) | 10,756 (96.8%) | <0.001 | 165,574 (99.0%) | 11,909 (96.9%) | <0.001 | 90,296 (99.1%) | 7,136 (97.0%) | <0.001 |
| Yes | 1,427 (1.0%) | 352 (3.2%) |  | 1,663 (1.0%) | 387 (3.1%) |  | 853 (0.9%) | 221 (3.0%) |  |
| Have you ever been diagnosed with diabetes mellitus? |  |  |  |  |  |  |  |  |  |
| No | 145,473 (98.2%) | 10,661 (96.0%) | <0.001 | 164,206 (98.2%) | 11,833 (96.2%) | <0.001 | 89,588 (98.3%) | 7,072 (96.1%) | <0.001 |
| Yes | 2,676 (1.8%) | 447 (4.0%) |  | 3,031 (1.8%) | 463 (3.8%) |  | 1,561 (1.7%) | 285 (3.9%) |  |
| Have you ever been diagnosed with heart disease? |  |  |  |  |  |  |  |  |  |
| No | 145,754 (98.4%) | 10,715 (96.5%) | <0.001 | 164,539 (98.4%) | 11,865 (96.5%) | <0.001 | 89,746 (98.5%) | 7,125 (96.8%) | <0.001 |
| Yes | 2,395 (1.6%) | 393 (3.5%) |  | 2,698 (1.6%) | 431 (3.5%) |  | 1,403 (1.5%) | 232 (3.2%) |  |
| Have you ever been diagnosed with asthma? |  |  |  |  |  |  |  |  |  |
| No | 144,441 (97.5%) | 10,429 (93.9%) | <0.001 | 163,012 (97.5%) | 11,558 (94.0%) | <0.001 | 88,950 (97.6%) | 6,910 (93.9%) | <0.001 |
| Yes | 3,708 (2.5%) | 679 (6.1%) |  | 4,225 (2.5%) | 738 (6.0%) |  | 2,199 (2.4%) | 447 (6.1%) |  |
| Have you ever been diagnosed with rheumatoid arthritis? |  |  |  |  |  |  |  |  |  |
| No | 137,114 (92.6%) | 9,489 (85.4%) | <0.001 | 154,867 (92.6%) | 10,553 (85.8%) | <0.001 | 84,471 (92.7%) | 6,322 (85.9%) | <0.001 |
| Yes | 11,035 (7.4%) | 1,619 (14.6%) |  | 12,370 (7.4%) | 1,743 (14.2%) |  | 6,678 (7.3%) | 1,035 (14.1%) |  |
| Have you ever been diagnosed with cancer? |  |  |  |  |  |  |  |  |  |
| No | 147,821 (99.8%) | 11,031 (99.3%) | <0.001 | 166,859 (99.8%) | 12,222 (99.4%) | <0.001 | 90,962 (99.8%) | 7,310 (99.4%) | <0.001 |
| Yes | 328 (0.2%) | 77 (0.7%) |  | 378 (0.2%) | 74 (0.6%) |  | 187 (0.2%) | 47 (0.6%) |  |
| Have you ever been diagnosed with kidney failure? |  |  |  |  |  |  |  |  |  |
| No | 147,624 (99.6%) | 10,992 (99.0%) | <0.001 | 166,624 (99.6%) | 12,178 (99.0%) | <0.001 | 90,805 (99.6%) | 7,279 (98.9%) | <0.001 |
| Yes | 525 (0.4%) | 116 (1.0%) |  | 613 (0.4%) | 118 (1.0%) |  | 344 (0.4%) | 78 (1.1%) |  |
| Family member with psychosis |  |  |  |  |  |  |  |  |  |
| No | 146,912 (99.2%) | 10,891 (98.0%) | <0.001 | 165,909 (99.2%) | 12,064 (98.1%) | <0.001 | 90,467 (99.3%) | 7,221 (98.2%) | <0.001 |
| Yes | 1,237 (0.8%) | 217 (2.0%) |  | 1,328 (0.8%) | 232 (1.9%) |  | 682 (0.7%) | 136 (1.8%) |  |
| Have difficulty accessing healthcare |  |  |  |  |  |  |  |  |  |
| No | 110,744 (85.8%) | 7,278 (79.3%) | <0.001 | 124,604 (85.7%) | 7,912 (78.4%) | <0.001 | 66,489 (84.7%) | 4,654 (77.6%) | <0.001 |
| Yes | 18,359 (14.2%) | 1,905 (20.7%) |  | 20,795 (14.3%) | 2,177 (21.6%) |  | 12,013 (15.3%) | 1,342 (22.4%) |  |

**Supplementary Table 3** Multivariable logistic regression results showing the associations between living in sea level rise districts and depression. *Source*: Riskesdas 2018 and Podes 2018.

| **Variables** | **Model 1** | | | | **Model 2** | | | | **Model 3** | | | | **Model 4** | | | |
| --- | --- | --- | --- | --- | --- | --- | --- | --- | --- | --- | --- | --- | --- | --- | --- | --- |
|  | **OR** | **P-Value** | **95% CI** | | **OR** | **P-Value** | **95% CI** | | **OR** | **P-Value** | **95% CI** | | **OR** | **P-Value** | **95% CI** | |
|  |  |  | **Lower** | **Upper** |  |  | **Lower** | **Upper** |  |  | **Lower** | **Upper** |  |  | **Lower** | **Upper** |
| Live at sea level rise districts | 1.16 | 0.000 | 1.14 | 1.19 | 1.15 | 0.000 | 1.13 | 1.18 | 1.16 | 0.000 | 1.14 | 1.19 | 1.13 | 0.000 | 1.10 | 1.16 |
| Age groups (Reference = 18-24 years old ) |  |  |  |  |  |  |  |  |  |  |  |  |  |  |  |  |
| 25-34 years old | 0.92 | 0.000 | 0.88 | 0.96 | 0.92 | 0.000 | 0.88 | 0.96 | 0.88 | 0.000 | 0.85 | 0.92 | 0.88 | 0.000 | 0.83 | 0.92 |
| 35-44 years old | 1.01 | 0.656 | 0.97 | 1.06 | 1.01 | 0.684 | 0.96 | 1.06 | 0.92 | 0.000 | 0.88 | 0.96 | 0.92 | 0.001 | 0.87 | 0.96 |
| 45-54 years old | 1.11 | 0.000 | 1.06 | 1.16 | 1.11 | 0.000 | 1.06 | 1.16 | 0.90 | 0.000 | 0.86 | 0.94 | 0.90 | 0.000 | 0.86 | 0.95 |
| 55-64 years old | 1.03 | 0.284 | 0.98 | 1.08 | 1.03 | 0.253 | 0.98 | 1.08 | 0.75 | 0.000 | 0.71 | 0.79 | 0.74 | 0.000 | 0.70 | 0.78 |
| 65-74 years old | 1.05 | 0.073 | 1.00 | 1.12 | 1.06 | 0.060 | 1.00 | 1.12 | 0.73 | 0.000 | 0.69 | 0.78 | 0.71 | 0.000 | 0.67 | 0.76 |
| 75 years old and above | 0.98 | 0.627 | 0.92 | 1.05 | 0.99 | 0.692 | 0.92 | 1.06 | 0.71 | 0.000 | 0.66 | 0.76 | 0.68 | 0.000 | 0.63 | 0.73 |
| Female (Reference = Male) | 1.41 | 0.000 | 1.38 | 1.45 | 2.12 | 0.000 | 2.05 | 2.20 | 2.15 | 0.000 | 2.08 | 2.23 | 2.17 | 0.000 | 2.08 | 2.25 |
| Education (Reference= Senior high school and higher) |  |  |  |  |  |  |  |  |  |  |  |  |  |  |  |  |
| Junior high school | 1.22 | 0.000 | 1.18 | 1.26 | 1.21 | 0.000 | 1.18 | 1.26 | 1.19 | 0.000 | 1.15 | 1.23 | 1.19 | 0.000 | 1.15 | 1.23 |
| Primary school and lower | 1.46 | 0.000 | 1.42 | 1.51 | 1.46 | 0.000 | 1.42 | 1.50 | 1.43 | 0.000 | 1.39 | 1.47 | 1.38 | 0.000 | 1.34 | 1.43 |
| Marital Status (Reference=Unmarried) |  |  |  |  |  |  |  |  |  |  |  |  |  |  |  |  |
| Married | 0.78 | 0.000 | 0.75 | 0.81 | 0.78 | 0.000 | 0.75 | 0.81 | 0.79 | 0.000 | 0.75 | 0.82 | 0.78 | 0.000 | 0.75 | 0.81 |
| Divorced and widowed | 1.16 | 0.000 | 1.10 | 1.22 | 1.14 | 0.000 | 1.08 | 1.20 | 1.13 | 0.000 | 1.08 | 1.19 | 1.13 | 0.000 | 1.07 | 1.19 |
| Employment Status (Reference=Jobless) |  |  |  |  |  |  |  |  |  |  |  |  |  |  |  |  |
| Student | 0.92 | 0.012 | 0.86 | 0.98 | 0.95 | 0.118 | 0.89 | 1.01 | 1.00 | 0.927 | 0.94 | 1.07 | 0.99 | 0.861 | 0.92 | 1.07 |
| Employee and retirement | 0.62 | 0.000 | 0.59 | 0.65 | 0.62 | 0.000 | 0.59 | 0.65 | 0.66 | 0.000 | 0.63 | 0.69 | 0.66 | 0.000 | 0.63 | 0.70 |
| Self-employee | 0.75 | 0.000 | 0.72 | 0.78 | 0.75 | 0.000 | 0.72 | 0.78 | 0.80 | 0.000 | 0.77 | 0.83 | 0.82 | 0.000 | 0.78 | 0.85 |
| Informal worker | 0.81 | 0.000 | 0.78 | 0.83 | 0.79 | 0.000 | 0.77 | 0.82 | 0.86 | 0.000 | 0.84 | 0.89 | 0.84 | 0.000 | 0.82 | 0.87 |
| Others | 0.84 | 0.000 | 0.81 | 0.88 | 0.84 | 0.000 | 0.80 | 0.88 | 0.88 | 0.000 | 0.84 | 0.92 | 0.87 | 0.000 | 0.83 | 0.92 |
| Household expenditure per month (Reference= 1st quintile) |  |  |  |  |  |  |  |  |  |  |  |  |  |  |  |  |
| 2nd quintile | 0.98 | 0.186 | 0.95 | 1.01 | 0.98 | 0.207 | 0.95 | 1.01 | 0.96 | 0.029 | 0.93 | 1.00 | 0.98 | 0.346 | 0.95 | 1.02 |
| 3rd quintile | 0.96 | 0.015 | 0.93 | 0.99 | 0.96 | 0.010 | 0.93 | 0.99 | 0.94 | 0.000 | 0.91 | 0.97 | 0.96 | 0.042 | 0.93 | 1.00 |
| 4th quintile | 0.89 | 0.000 | 0.86 | 0.92 | 0.89 | 0.000 | 0.86 | 0.92 | 0.86 | 0.000 | 0.83 | 0.89 | 0.89 | 0.000 | 0.86 | 0.92 |
| 5th quintile | 0.80 | 0.000 | 0.77 | 0.83 | 0.79 | 0.000 | 0.76 | 0.82 | 0.75 | 0.000 | 0.72 | 0.78 | 0.78 | 0.000 | 0.75 | 0.81 |
| Smoking status (Reference = Not Smokers) |  |  |  |  |  |  |  |  |  |  |  |  |  |  |  |  |
| Not everyday |  |  |  |  | 1.03 | 0.274 | 0.98 | 1.09 | 1.01 | 0.739 | 0.96 | 1.07 | 1.00 | 0.895 | 0.94 | 1.06 |
| Ex smoker |  |  |  |  | 1.44 | 0.000 | 1.37 | 1.51 | 1.24 | 0.000 | 1.18 | 1.30 | 1.24 | 0.000 | 1.18 | 1.31 |
| Not smoker |  |  |  |  | 0.66 | 0.000 | 0.63 | 0.68 | 0.62 | 0.000 | 0.60 | 0.65 | 0.62 | 0.000 | 0.60 | 0.65 |
| Alcohol use (Reference = Under standard) |  |  |  |  |  |  |  |  |  |  |  |  |  |  |  |  |
| More than standard |  |  |  |  | 0.99 | 0.773 | 0.91 | 1.07 | 0.99 | 0.757 | 0.91 | 1.07 | 0.97 | 0.519 | 0.89 | 1.06 |
| No alcohol |  |  |  |  | 0.56 | 0.000 | 0.53 | 0.59 | 0.56 | 0.000 | 0.53 | 0.59 | 0.55 | 0.000 | 0.52 | 0.59 |
| Active physical activity (Reference = Less active) |  |  |  |  | 0.89 | 0.000 | 0.86 | 0.92 | 0.90 | 0.000 | 0.87 | 0.93 | 0.90 | 0.000 | 0.87 | 0.94 |
| Have you ever been diagnosed with lung tuberculosis |  |  |  |  |  |  |  |  | 2.16 | 0.000 | 1.95 | 2.40 | 2.20 | 0.000 | 1.97 | 2.45 |
| Have you ever been diagnosed with hypertension |  |  |  |  |  |  |  |  | 1.41 | 0.000 | 1.36 | 1.46 | 1.42 | 0.000 | 1.37 | 1.47 |
| Have you ever been diagnosed with stroke |  |  |  |  |  |  |  |  | 2.39 | 0.000 | 2.24 | 2.55 | 2.45 | 0.000 | 2.29 | 2.62 |
| Have you ever been diagnosed with diabetes mellitus |  |  |  |  |  |  |  |  | 1.62 | 0.000 | 1.53 | 1.71 | 1.64 | 0.000 | 1.54 | 1.73 |
| Have you ever been diagnosed with heart disease |  |  |  |  |  |  |  |  | 1.56 | 0.000 | 1.47 | 1.65 | 1.57 | 0.000 | 1.48 | 1.67 |
| Have you ever been diagnosed with asthma |  |  |  |  |  |  |  |  | 2.01 | 0.000 | 1.92 | 2.11 | 2.02 | 0.000 | 1.92 | 2.13 |
| Have you ever been diagnosed with rheumatoid arthritis |  |  |  |  |  |  |  |  | 1.81 | 0.000 | 1.76 | 1.87 | 1.85 | 0.000 | 1.79 | 1.91 |
| Have you ever been diagnosed with cancer |  |  |  |  |  |  |  |  | 2.24 | 0.000 | 1.96 | 2.57 | 2.33 | 0.000 | 2.02 | 2.69 |
| Have you ever been diagnosed with kidney failure |  |  |  |  |  |  |  |  | 2.12 | 0.000 | 1.90 | 2.37 | 2.08 | 0.000 | 1.85 | 2.34 |
| Having families with psychosis |  |  |  |  |  |  |  |  | 2.36 | 0.000 | 2.19 | 2.56 | 2.33 | 0.000 | 2.14 | 2.53 |
| Have difficulty to access healthcare |  |  |  |  |  |  |  |  |  |  |  |  | 1.37 | 0.000 | 1.33 | 1.42 |
| Constant | 0.12 | 0.000 | 0.11 | 0.13 | 0.12 | 0.000 | 0.11 | 0.13 | 0.11 | 0.000 | 0.10 | 0.12 | 0.11 | 0.000 | 0.10 | 0.12 |

**Notes:** Model 1=adjusted with demographic and socioeconomics variables; Model 2=adjusted with demographic, socioeconomic, and lifestyle variables; Model 3=adjusted with demographic, socioeconomic, lifestyle and the presence of comorbidities variables; Model 4=adjusted with demographic, socioeconomic, lifestyle, the presence of comorbidities and access to healthcare variables. OR=Odds ratio; P=P-value; 95%CI=95% Confidence Intervals.

**Supplementary Table 4** Multivariable logistic regression results showing the associations between living in districts affected by coastal abrassion and depression. *Source*: Riskesdas 2018 and Podes 2018.

| **Variables** | **Model 1** | | | | **Model 2** | | | | **Model 3** | | | | **Model 4** | | | |
| --- | --- | --- | --- | --- | --- | --- | --- | --- | --- | --- | --- | --- | --- | --- | --- | --- |
|  | **OR** | **P-Value** | **95% CI** | | **OR** | **P-Value** | **95% CI** | | **OR** | **P-Value** | **95% CI** | | **OR** | **P-Value** | **95% CI** | |
|  |  |  | **Lower** | **Upper** |  |  | **Lower** | **Upper** |  |  | **Lower** | **Upper** |  |  | **Lower** | **Upper** |
| Live at coastal abrasion area | 1.19 | 0.000 | 1.16 | 1.22 | 1.17 | 0.000 | 1.15 | 1.20 | 1.19 | 0.000 | 1.16 | 1.22 | 1.16 | 0.000 | 1.13 | 1.19 |
| Age groups (Reference = 18-24 years old ) |  |  |  |  |  |  |  |  |  |  |  |  |  |  |  |  |
| 25-34 years old | 0.92 | 0.000 | 0.88 | 0.96 | 0.92 | 0.000 | 0.88 | 0.96 | 0.88 | 0.000 | 0.85 | 0.92 | 0.88 | 0.000 | 0.84 | 0.92 |
| 35-44 years old | 1.01 | 0.618 | 0.97 | 1.06 | 1.01 | 0.648 | 0.97 | 1.06 | 0.92 | 0.000 | 0.88 | 0.96 | 0.92 | 0.001 | 0.87 | 0.96 |
| 45-54 years old | 1.11 | 0.000 | 1.06 | 1.16 | 1.11 | 0.000 | 1.06 | 1.16 | 0.90 | 0.000 | 0.86 | 0.94 | 0.90 | 0.000 | 0.86 | 0.95 |
| 55-64 years old | 1.03 | 0.257 | 0.98 | 1.08 | 1.03 | 0.231 | 0.98 | 1.08 | 0.75 | 0.000 | 0.71 | 0.79 | 0.74 | 0.000 | 0.70 | 0.78 |
| 65-74 years old | 1.06 | 0.059 | 1.00 | 1.12 | 1.06 | 0.050 | 1.00 | 1.12 | 0.74 | 0.000 | 0.69 | 0.78 | 0.72 | 0.000 | 0.67 | 0.76 |
| 75 years old and above | 0.99 | 0.688 | 0.92 | 1.06 | 0.99 | 0.748 | 0.92 | 1.06 | 0.71 | 0.000 | 0.66 | 0.76 | 0.68 | 0.000 | 0.63 | 0.74 |
| Female (Reference = Male) | 1.41 | 0.000 | 1.38 | 1.45 | 2.12 | 0.000 | 2.05 | 2.20 | 2.15 | 0.000 | 2.08 | 2.23 | 2.16 | 0.000 | 2.08 | 2.25 |
| Education (Reference= Senior high school and higher) |  |  |  |  |  |  |  |  |  |  |  |  |  |  |  |  |
| Junior high school | 1.22 | 0.000 | 1.18 | 1.26 | 1.21 | 0.000 | 1.17 | 1.26 | 1.19 | 0.000 | 1.15 | 1.23 | 1.19 | 0.000 | 1.14 | 1.23 |
| Primary school and lower | 1.46 | 0.000 | 1.42 | 1.50 | 1.46 | 0.000 | 1.41 | 1.50 | 1.43 | 0.000 | 1.39 | 1.47 | 1.38 | 0.000 | 1.34 | 1.43 |
| Marital Status (Reference=Unmarried) |  |  |  |  |  |  |  |  |  |  |  |  |  |  |  |  |
| Married | 0.78 | 0.000 | 0.75 | 0.81 | 0.78 | 0.000 | 0.75 | 0.81 | 0.78 | 0.000 | 0.75 | 0.82 | 0.78 | 0.000 | 0.75 | 0.81 |
| Divorced and widowed | 1.16 | 0.000 | 1.10 | 1.22 | 1.14 | 0.000 | 1.08 | 1.20 | 1.13 | 0.000 | 1.07 | 1.19 | 1.13 | 0.000 | 1.06 | 1.19 |
| Employment Status (Reference=Jobless) |  |  |  |  |  |  |  |  |  |  |  |  |  |  |  |  |
| Student | 0.92 | 0.015 | 0.86 | 0.98 | 0.95 | 0.136 | 0.89 | 1.02 | 1.01 | 0.862 | 0.94 | 1.08 | 1.00 | 0.908 | 0.93 | 1.07 |
| Employee and retirement | 0.62 | 0.000 | 0.59 | 0.65 | 0.62 | 0.000 | 0.59 | 0.65 | 0.66 | 0.000 | 0.63 | 0.69 | 0.67 | 0.000 | 0.64 | 0.70 |
| Self employee | 0.75 | 0.000 | 0.73 | 0.78 | 0.75 | 0.000 | 0.73 | 0.78 | 0.80 | 0.000 | 0.77 | 0.83 | 0.82 | 0.000 | 0.79 | 0.85 |
| Informal worker | 0.81 | 0.000 | 0.78 | 0.83 | 0.79 | 0.000 | 0.77 | 0.82 | 0.87 | 0.000 | 0.84 | 0.89 | 0.84 | 0.000 | 0.82 | 0.87 |
| Others | 0.84 | 0.000 | 0.81 | 0.88 | 0.84 | 0.000 | 0.80 | 0.88 | 0.88 | 0.000 | 0.84 | 0.92 | 0.87 | 0.000 | 0.83 | 0.91 |
| Household expenditure per month (Reference= 1st quintile) |  |  |  |  |  |  |  |  |  |  |  |  |  |  |  |  |
| 2nd quintile | 0.98 | 0.196 | 0.95 | 1.01 | 0.98 | 0.220 | 0.95 | 1.01 | 0.97 | 0.032 | 0.93 | 1.00 | 0.98 | 0.363 | 0.95 | 1.02 |
| 3rd quintile | 0.96 | 0.016 | 0.93 | 0.99 | 0.96 | 0.011 | 0.93 | 0.99 | 0.94 | 0.000 | 0.91 | 0.97 | 0.96 | 0.047 | 0.93 | 1.00 |
| 4th quintile | 0.89 | 0.000 | 0.86 | 0.92 | 0.89 | 0.000 | 0.86 | 0.92 | 0.86 | 0.000 | 0.83 | 0.89 | 0.89 | 0.000 | 0.86 | 0.92 |
| 5th quintile | 0.80 | 0.000 | 0.77 | 0.83 | 0.79 | 0.000 | 0.77 | 0.82 | 0.75 | 0.000 | 0.72 | 0.78 | 0.79 | 0.000 | 0.76 | 0.82 |
| Smoking status (Reference = Not Smokers) |  |  |  |  |  |  |  |  |  |  |  |  |  |  |  |  |
| Not everyday |  |  |  |  | 1.03 | 0.254 | 0.98 | 1.09 | 1.01 | 0.703 | 0.96 | 1.07 | 1.00 | 0.923 | 0.94 | 1.06 |
| Ex smoker |  |  |  |  | 1.44 | 0.000 | 1.37 | 1.51 | 1.24 | 0.000 | 1.18 | 1.30 | 1.25 | 0.000 | 1.18 | 1.31 |
| Not smoker |  |  |  |  | 0.66 | 0.000 | 0.63 | 0.68 | 0.63 | 0.000 | 0.60 | 0.65 | 0.62 | 0.000 | 0.60 | 0.65 |
| Alcohol use (Reference = Under standard) |  |  |  |  |  |  |  |  |  |  |  |  |  |  |  |  |
| More than standard |  |  |  |  | 0.99 | 0.761 | 0.91 | 1.07 | 0.99 | 0.746 | 0.91 | 1.07 | 0.97 | 0.510 | 0.89 | 1.06 |
| No alcohol |  |  |  |  | 0.56 | 0.000 | 0.53 | 0.59 | 0.56 | 0.000 | 0.53 | 0.59 | 0.55 | 0.000 | 0.52 | 0.58 |
| Active physical activity (Reference = Less active) |  |  |  |  | 0.90 | 0.000 | 0.87 | 0.93 | 0.90 | 0.000 | 0.87 | 0.94 | 0.91 | 0.000 | 0.87 | 0.94 |
| Have you ever been diagnosed with lung tubercolosis |  |  |  |  |  |  |  |  | 2.16 | 0.000 | 1.95 | 2.39 | 2.20 | 0.000 | 1.97 | 2.45 |
| Have you ever been diagnosed with hypertension |  |  |  |  |  |  |  |  | 1.41 | 0.000 | 1.37 | 1.46 | 1.42 | 0.000 | 1.37 | 1.47 |
| Have you ever been diagnosed with stroke |  |  |  |  |  |  |  |  | 2.39 | 0.000 | 2.24 | 2.55 | 2.45 | 0.000 | 2.29 | 2.62 |
| Have you ever been diagnosed with diabetes mellitus |  |  |  |  |  |  |  |  | 1.62 | 0.000 | 1.53 | 1.71 | 1.64 | 0.000 | 1.54 | 1.73 |
| Have you ever been diagnosed with heart disease |  |  |  |  |  |  |  |  | 1.56 | 0.000 | 1.47 | 1.65 | 1.57 | 0.000 | 1.48 | 1.67 |
| Have you ever been diagnosed with asthma |  |  |  |  |  |  |  |  | 2.01 | 0.000 | 1.92 | 2.11 | 2.02 | 0.000 | 1.92 | 2.13 |
| Have you ever been diagnosed with rheumatoid arthritis |  |  |  |  |  |  |  |  | 1.81 | 0.000 | 1.76 | 1.87 | 1.85 | 0.000 | 1.79 | 1.91 |
| Have you ever been diagnosed with cancer |  |  |  |  |  |  |  |  | 2.24 | 0.000 | 1.96 | 2.57 | 2.33 | 0.000 | 2.02 | 2.69 |
| Have you ever been diagnosed with kidney failure |  |  |  |  |  |  |  |  | 2.12 | 0.000 | 1.90 | 2.37 | 2.08 | 0.000 | 1.85 | 2.34 |
| Having families with psychosis |  |  |  |  |  |  |  |  | 2.36 | 0.000 | 2.19 | 2.56 | 2.33 | 0.000 | 2.14 | 2.53 |
| Have difficulty to access healthcare |  |  |  |  |  |  |  |  |  |  |  |  | 1.37 | 0.000 | 1.33 | 1.42 |
| Constant | 0.06 | 0.000 | 0.06 | 0.06 | 0.12 | 0.000 | 0.11 | 0.13 | 0.11 | 0.000 | 0.10 | 0.12 | 0.11 | 0.000 | 0.10 | 0.12 |

**Notes:** Model 1=adjusted with demographic and socioeconomics variables; Model 2=adjusted with demographic, socioeconomic, and lifestyle variables; Model 3=adjusted with demographic, socioeconomic, lifestyle and the presence of comorbidities variables; Model 4=adjusted with demographic, socioeconomic, lifestyle, the presence of comorbidities and access to healthcare variables. OR=Odds ratio; P=P-value; 95%CI=95% Confidence Intervals.

**Supplementary Table 5** Multivariable logistic regression results showing the associations between living in districts affected by hurricane and depression. *Source*: Riskesdas 2018 and Podes 2018.

| **Variables** | **Model 1** | | | | **Model 2** | | | | **Model 3** | | | | **Model 4** | | | |
| --- | --- | --- | --- | --- | --- | --- | --- | --- | --- | --- | --- | --- | --- | --- | --- | --- |
|  | **OR** | **P-Value** | **95% CI** | | **OR** | **P-Value** | **95% CI** | | **OR** | **P-Value** | **95% CI** | | **OR** | **P-Value** | **95% CI** | |
|  |  |  | **Lower** | **Upper** |  |  | **Lower** | **Upper** |  |  | **Lower** | **Upper** |  |  | **Lower** | **Upper** |
| Live at hurricane districts | 1.17 | 0.000 | 1.15 | 1.20 | 1.16 | 0.000 | 1.13 | 1.19 | 1.18 | 0.000 | 1.15 | 1.21 | 1.14 | 0.000 | 1.11 | 1.16 |
| Age groups (Reference = 18-24 years old ) |  |  |  |  |  |  |  |  |  |  |  |  |  |  |  |  |
| 25-34 years old | 0.92 | 0.000 | 0.88 | 0.96 | 0.92 | 0.000 | 0.88 | 0.96 | 0.88 | 0.000 | 0.85 | 0.92 | 0.88 | 0.000 | 0.84 | 0.92 |
| 35-44 years old | 1.01 | 0.642 | 0.97 | 1.06 | 1.01 | 0.670 | 0.97 | 1.06 | 0.92 | 0.000 | 0.88 | 0.96 | 0.92 | 0.001 | 0.87 | 0.96 |
| 45-54 years old | 1.11 | 0.000 | 1.06 | 1.16 | 1.11 | 0.000 | 1.06 | 1.16 | 0.90 | 0.000 | 0.86 | 0.94 | 0.90 | 0.000 | 0.86 | 0.95 |
| 55-64 years old | 1.03 | 0.282 | 0.98 | 1.08 | 1.03 | 0.252 | 0.98 | 1.08 | 0.75 | 0.000 | 0.71 | 0.79 | 0.74 | 0.000 | 0.70 | 0.78 |
| 65-74 years old | 1.05 | 0.072 | 1.00 | 1.12 | 1.06 | 0.060 | 1.00 | 1.12 | 0.73 | 0.000 | 0.69 | 0.78 | 0.71 | 0.000 | 0.67 | 0.76 |
| 75 years old and above | 0.98 | 0.620 | 0.92 | 1.05 | 0.99 | 0.686 | 0.92 | 1.06 | 0.71 | 0.000 | 0.66 | 0.76 | 0.68 | 0.000 | 0.63 | 0.73 |
| Female (Reference = Male) | 1.41 | 0.000 | 1.38 | 1.45 | 2.12 | 0.000 | 2.05 | 2.20 | 2.15 | 0.000 | 2.08 | 2.23 | 2.16 | 0.000 | 2.08 | 2.25 |
| Education (Reference= Senior high school and higher) |  |  |  |  |  |  |  |  |  |  |  |  |  |  |  |  |
| Junior high school | 1.22 | 0.000 | 1.18 | 1.26 | 1.21 | 0.000 | 1.17 | 1.26 | 1.19 | 0.000 | 1.15 | 1.23 | 1.19 | 0.000 | 1.14 | 1.23 |
| Primary school and lower | 1.46 | 0.000 | 1.42 | 1.50 | 1.46 | 0.000 | 1.42 | 1.50 | 1.43 | 0.000 | 1.39 | 1.47 | 1.38 | 0.000 | 1.34 | 1.43 |
| Marital Status (Reference=Unmarried) |  |  |  |  |  |  |  |  |  |  |  |  |  |  |  |  |
| Married | 0.78 | 0.000 | 0.75 | 0.81 | 0.78 | 0.000 | 0.75 | 0.81 | 0.79 | 0.000 | 0.75 | 0.82 | 0.78 | 0.000 | 0.75 | 0.81 |
| Divorced and widowed | 1.16 | 0.000 | 1.10 | 1.22 | 1.14 | 0.000 | 1.08 | 1.20 | 1.13 | 0.000 | 1.07 | 1.19 | 1.13 | 0.000 | 1.07 | 1.19 |
| Employment Status (Reference=Jobless) |  |  |  |  |  |  |  |  |  |  |  |  |  |  |  |  |
| Student | 0.92 | 0.012 | 0.86 | 0.98 | 0.95 | 0.112 | 0.89 | 1.01 | 1.00 | 0.956 | 0.94 | 1.07 | 0.99 | 0.844 | 0.92 | 1.07 |
| Employee and retirement | 0.62 | 0.000 | 0.59 | 0.64 | 0.62 | 0.000 | 0.59 | 0.65 | 0.66 | 0.000 | 0.63 | 0.69 | 0.66 | 0.000 | 0.63 | 0.70 |
| Self employee | 0.75 | 0.000 | 0.72 | 0.78 | 0.75 | 0.000 | 0.72 | 0.78 | 0.80 | 0.000 | 0.77 | 0.83 | 0.81 | 0.000 | 0.78 | 0.85 |
| Informal worker | 0.80 | 0.000 | 0.78 | 0.83 | 0.79 | 0.000 | 0.77 | 0.81 | 0.86 | 0.000 | 0.84 | 0.89 | 0.84 | 0.000 | 0.82 | 0.87 |
| Others | 0.84 | 0.000 | 0.81 | 0.88 | 0.84 | 0.000 | 0.80 | 0.88 | 0.88 | 0.000 | 0.84 | 0.92 | 0.87 | 0.000 | 0.83 | 0.92 |
| Household expenditure per month (Reference= 1st quintile) |  |  |  |  |  |  |  |  |  |  |  |  |  |  |  |  |
| 2nd quintile | 0.98 | 0.212 | 0.95 | 1.01 | 0.98 | 0.233 | 0.95 | 1.01 | 0.97 | 0.036 | 0.93 | 1.00 | 0.98 | 0.374 | 0.95 | 1.02 |
| 3rd quintile | 0.96 | 0.022 | 0.93 | 0.99 | 0.96 | 0.014 | 0.93 | 0.99 | 0.94 | 0.000 | 0.91 | 0.97 | 0.97 | 0.053 | 0.93 | 1.00 |
| 4th quintile | 0.90 | 0.000 | 0.87 | 0.93 | 0.89 | 0.000 | 0.86 | 0.92 | 0.86 | 0.000 | 0.83 | 0.89 | 0.89 | 0.000 | 0.86 | 0.92 |
| 5th quintile | 0.80 | 0.000 | 0.77 | 0.83 | 0.79 | 0.000 | 0.77 | 0.82 | 0.75 | 0.000 | 0.72 | 0.78 | 0.79 | 0.000 | 0.76 | 0.82 |
| Smoking status (Reference = Not Smokers) |  |  |  |  |  |  |  |  |  |  |  |  |  |  |  |  |
| Not everyday |  |  |  |  | 1.03 | 0.272 | 0.98 | 1.09 | 1.01 | 0.736 | 0.96 | 1.07 | 1.00 | 0.902 | 0.94 | 1.06 |
| Ex smoker |  |  |  |  | 1.44 | 0.000 | 1.37 | 1.51 | 1.24 | 0.000 | 1.18 | 1.30 | 1.24 | 0.000 | 1.18 | 1.31 |
| Not smoker |  |  |  |  | 0.66 | 0.000 | 0.63 | 0.68 | 0.62 | 0.000 | 0.60 | 0.65 | 0.62 | 0.000 | 0.60 | 0.65 |
| Alcohol use (Reference = Under standard) |  |  |  |  |  |  |  |  |  |  |  |  |  |  |  |  |
| More than standard |  |  |  |  | 0.99 | 0.802 | 0.91 | 1.08 | 0.99 | 0.779 | 0.91 | 1.07 | 0.97 | 0.547 | 0.89 | 1.07 |
| No alcohol |  |  |  |  | 0.56 | 0.000 | 0.53 | 0.59 | 0.56 | 0.000 | 0.53 | 0.59 | 0.55 | 0.000 | 0.52 | 0.59 |
| Active physical activity (Reference = Less active) |  |  |  |  | 0.89 | 0.000 | 0.86 | 0.92 | 0.90 | 0.000 | 0.87 | 0.93 | 0.90 | 0.000 | 0.87 | 0.94 |
| Have you ever been diagnosed with lung tubercolosis |  |  |  |  |  |  |  |  | 2.16 | 0.000 | 1.95 | 2.40 | 2.20 | 0.000 | 1.97 | 2.45 |
| Have you ever been diagnosed with hypertension |  |  |  |  |  |  |  |  | 1.41 | 0.000 | 1.37 | 1.46 | 1.42 | 0.000 | 1.37 | 1.47 |
| Have you ever been diagnosed with stroke |  |  |  |  |  |  |  |  | 2.39 | 0.000 | 2.24 | 2.55 | 2.45 | 0.000 | 2.29 | 2.62 |
| Have you ever been diagnosed with diabetes mellitus |  |  |  |  |  |  |  |  | 1.62 | 0.000 | 1.54 | 1.71 | 1.64 | 0.000 | 1.54 | 1.73 |
| Have you ever been diagnosed with heart disease |  |  |  |  |  |  |  |  | 1.56 | 0.000 | 1.47 | 1.65 | 1.57 | 0.000 | 1.48 | 1.67 |
| Have you ever been diagnosed with asthma |  |  |  |  |  |  |  |  | 2.01 | 0.000 | 1.92 | 2.11 | 2.02 | 0.000 | 1.92 | 2.13 |
| Have you ever been diagnosed with rheumatoid arthritis |  |  |  |  |  |  |  |  | 1.81 | 0.000 | 1.76 | 1.87 | 1.85 | 0.000 | 1.79 | 1.91 |
| Have you ever been diagnosed with cancer |  |  |  |  |  |  |  |  | 2.24 | 0.000 | 1.96 | 2.57 | 2.34 | 0.000 | 2.03 | 2.69 |
| Have you ever been diagnosed with kidney failure |  |  |  |  |  |  |  |  | 2.12 | 0.000 | 1.90 | 2.37 | 2.08 | 0.000 | 1.85 | 2.34 |
| Having families with psychosis |  |  |  |  |  |  |  |  | 2.37 | 0.000 | 2.19 | 2.56 | 2.33 | 0.000 | 2.14 | 2.54 |
| Have difficulty to access healthcare |  |  |  |  |  |  |  |  |  |  |  |  | 1.37 | 0.000 | 1.33 | 1.41 |
| Constant | 0.06 | 0.000 | 0.06 | 0.06 | 0.12 | 0.000 | 0.11 | 0.13 | 0.11 | 0.000 | 0.10 | 0.12 | 0.11 | 0.000 | 0.10 | 0.12 |

**Notes:** Model 1=adjusted with demographic and socioeconomics variables; Model 2=adjusted with demographic, socioeconomic, and lifestyle variables; Model 3=adjusted with demographic, socioeconomic, lifestyle and the presence of comorbidities variables; Model 4=adjusted with demographic, socioeconomic, lifestyle, the presence of comorbidities and access to healthcare variables. OR=Odds ratio; P=P-value; 95%CI=95% Confidence Intervals.

**Supplementary Table 6** Multivariable logistic regression results showing the associations between living in districts affected by coastal tidal flooding and depression. *Source*: Riskesdas 2018 and Podes 2018.

| **Variables** | **Model 1** | | | | **Model 2** | | | | **Model 3** | | | | **Model 4** | | | |
| --- | --- | --- | --- | --- | --- | --- | --- | --- | --- | --- | --- | --- | --- | --- | --- | --- |
|  | **OR** | **P-Value** | **95% CI** | | **OR** | **P-Value** | **95% CI** | | **OR** | **P-Value** | **95% CI** | | **OR** | **P-Value** | **95% CI** | |
|  |  |  | **Lower** | **Upper** |  |  | **Lower** | **Upper** |  |  | **Lower** | **Upper** |  |  | **Lower** | **Upper** |
| Live at coastal tidal flooding district | 1.27 | 0.000 | 1.23 | 1.30 | 1.26 | 0.000 | 1.22 | 1.29 | 1.28 | 0.000 | 1.24 | 1.31 | 1.24 | 0.000 | 1.20 | 1.28 |
| Age groups (Reference = 18-24 years old ) |  |  |  |  |  |  |  |  |  |  |  |  |  |  |  |  |
| 25-34 years old | 0.92 | 0.000 | 0.88 | 0.96 | 0.92 | 0.000 | 0.88 | 0.96 | 0.89 | 0.000 | 0.85 | 0.93 | 0.88 | 0.000 | 0.84 | 0.92 |
| 35-44 years old | 1.01 | 0.586 | 0.97 | 1.06 | 1.01 | 0.603 | 0.97 | 1.06 | 0.92 | 0.000 | 0.88 | 0.96 | 0.92 | 0.001 | 0.87 | 0.96 |
| 45-54 years old | 1.11 | 0.000 | 1.06 | 1.16 | 1.11 | 0.000 | 1.06 | 1.17 | 0.90 | 0.000 | 0.86 | 0.95 | 0.90 | 0.000 | 0.86 | 0.95 |
| 55-64 years old | 1.03 | 0.243 | 0.98 | 1.08 | 1.03 | 0.213 | 0.98 | 1.09 | 0.75 | 0.000 | 0.71 | 0.79 | 0.74 | 0.000 | 0.70 | 0.78 |
| 65-74 years old | 1.06 | 0.057 | 1.00 | 1.12 | 1.06 | 0.047 | 1.00 | 1.12 | 0.74 | 0.000 | 0.69 | 0.78 | 0.72 | 0.000 | 0.67 | 0.76 |
| 75 years old and above | 0.99 | 0.708 | 0.92 | 1.06 | 0.99 | 0.774 | 0.92 | 1.06 | 0.71 | 0.000 | 0.66 | 0.76 | 0.68 | 0.000 | 0.63 | 0.74 |
| Female (Reference = Male) | 1.41 | 0.000 | 1.38 | 1.45 | 2.12 | 0.000 | 2.04 | 2.20 | 2.15 | 0.000 | 2.07 | 2.23 | 2.16 | 0.000 | 2.08 | 2.25 |
| Education (Reference= Senior high school and higher) |  |  |  |  |  |  |  |  |  |  |  |  |  |  |  |  |
| Junior high school | 1.22 | 0.000 | 1.18 | 1.26 | 1.21 | 0.000 | 1.17 | 1.25 | 1.19 | 0.000 | 1.15 | 1.23 | 1.18 | 0.000 | 1.14 | 1.23 |
| Primary school and lower | 1.46 | 0.000 | 1.42 | 1.50 | 1.46 | 0.000 | 1.41 | 1.50 | 1.43 | 0.000 | 1.38 | 1.47 | 1.38 | 0.000 | 1.34 | 1.43 |
| Marital Status (Reference=Unmarried) |  |  |  |  |  |  |  |  |  |  |  |  |  |  |  |  |
| Married | 0.78 | 0.000 | 0.75 | 0.81 | 0.78 | 0.000 | 0.75 | 0.81 | 0.78 | 0.000 | 0.75 | 0.82 | 0.78 | 0.000 | 0.75 | 0.81 |
| Divorced and widowed | 1.15 | 0.000 | 1.10 | 1.21 | 1.14 | 0.000 | 1.08 | 1.20 | 1.13 | 0.000 | 1.07 | 1.19 | 1.12 | 0.000 | 1.06 | 1.19 |
| Employment Status (Reference=Jobless) |  |  |  |  |  |  |  |  |  |  |  |  |  |  |  |  |
| Student | 0.92 | 0.012 | 0.86 | 0.98 | 0.95 | 0.114 | 0.89 | 1.01 | 1.00 | 0.947 | 0.94 | 1.07 | 0.99 | 0.846 | 0.92 | 1.07 |
| Employee and retirement | 0.62 | 0.000 | 0.59 | 0.65 | 0.62 | 0.000 | 0.59 | 0.65 | 0.66 | 0.000 | 0.63 | 0.69 | 0.67 | 0.000 | 0.63 | 0.70 |
| Self-employee | 0.75 | 0.000 | 0.72 | 0.78 | 0.75 | 0.000 | 0.72 | 0.78 | 0.80 | 0.000 | 0.77 | 0.83 | 0.81 | 0.000 | 0.78 | 0.85 |
| Informal worker | 0.80 | 0.000 | 0.78 | 0.83 | 0.79 | 0.000 | 0.77 | 0.81 | 0.86 | 0.000 | 0.84 | 0.89 | 0.84 | 0.000 | 0.82 | 0.87 |
| Others | 0.84 | 0.000 | 0.81 | 0.88 | 0.84 | 0.000 | 0.80 | 0.88 | 0.88 | 0.000 | 0.84 | 0.92 | 0.87 | 0.000 | 0.83 | 0.92 |
| Household expenditure per month (Reference= 1st quintile) |  |  |  |  |  |  |  |  |  |  |  |  |  |  |  |  |
| 2nd quintile | 0.98 | 0.199 | 0.95 | 1.01 | 0.98 | 0.228 | 0.95 | 1.01 | 0.97 | 0.034 | 0.93 | 1.00 | 0.98 | 0.401 | 0.95 | 1.02 |
| 3rd quintile | 0.96 | 0.020 | 0.93 | 0.99 | 0.96 | 0.014 | 0.93 | 0.99 | 0.94 | 0.000 | 0.91 | 0.97 | 0.97 | 0.062 | 0.93 | 1.00 |
| 4th quintile | 0.90 | 0.000 | 0.87 | 0.93 | 0.89 | 0.000 | 0.86 | 0.92 | 0.86 | 0.000 | 0.83 | 0.89 | 0.89 | 0.000 | 0.86 | 0.92 |
| 5th quintile | 0.80 | 0.000 | 0.78 | 0.83 | 0.80 | 0.000 | 0.77 | 0.82 | 0.75 | 0.000 | 0.73 | 0.78 | 0.79 | 0.000 | 0.76 | 0.82 |
| Smoking status (Reference = Not Smokers) |  |  |  |  |  |  |  |  |  |  |  |  |  |  |  |  |
| Not everyday |  |  |  |  | 1.03 | 0.255 | 0.98 | 1.09 | 1.01 | 0.700 | 0.96 | 1.07 | 1.00 | 0.941 | 0.94 | 1.06 |
| Ex smoker |  |  |  |  | 1.44 | 0.000 | 1.38 | 1.51 | 1.25 | 0.000 | 1.19 | 1.31 | 1.25 | 0.000 | 1.19 | 1.32 |
| Not smoker |  |  |  |  | 0.66 | 0.000 | 0.63 | 0.69 | 0.63 | 0.000 | 0.60 | 0.65 | 0.62 | 0.000 | 0.60 | 0.65 |
| Alcohol use (Reference = Under standard) |  |  |  |  |  |  |  |  |  |  |  |  |  |  |  |  |
| More than standard |  |  |  |  | 0.99 | 0.836 | 0.91 | 1.08 | 0.99 | 0.815 | 0.91 | 1.08 | 0.97 | 0.513 | 0.89 | 1.06 |
| No alcohol |  |  |  |  | 0.56 | 0.000 | 0.52 | 0.59 | 0.56 | 0.000 | 0.53 | 0.59 | 0.55 | 0.000 | 0.51 | 0.58 |
| Active physical activity (Reference = Less active) |  |  |  |  | 0.89 | 0.000 | 0.86 | 0.92 | 0.90 | 0.000 | 0.87 | 0.93 | 0.90 | 0.000 | 0.87 | 0.94 |
| Have you ever been diagnosed with lung tuberculosis |  |  |  |  |  |  |  |  | 2.16 | 0.000 | 1.95 | 2.39 | 2.20 | 0.000 | 1.97 | 2.45 |
| Have you ever been diagnosed with hypertension |  |  |  |  |  |  |  |  | 1.41 | 0.000 | 1.36 | 1.46 | 1.42 | 0.000 | 1.37 | 1.47 |
| Have you ever been diagnosed with stroke |  |  |  |  |  |  |  |  | 2.39 | 0.000 | 2.24 | 2.55 | 2.45 | 0.000 | 2.29 | 2.62 |
| Have you ever been diagnosed with diabetes mellitus |  |  |  |  |  |  |  |  | 1.62 | 0.000 | 1.54 | 1.71 | 1.64 | 0.000 | 1.54 | 1.73 |
| Have you ever been diagnosed with heart disease |  |  |  |  |  |  |  |  | 1.56 | 0.000 | 1.47 | 1.65 | 1.58 | 0.000 | 1.48 | 1.68 |
| Have you ever been diagnosed with asthma |  |  |  |  |  |  |  |  | 2.01 | 0.000 | 1.92 | 2.11 | 2.03 | 0.000 | 1.93 | 2.13 |
| Have you ever been diagnosed with rheumatoid arthritis |  |  |  |  |  |  |  |  | 1.81 | 0.000 | 1.76 | 1.87 | 1.85 | 0.000 | 1.79 | 1.91 |
| Have you ever been diagnosed with cancer |  |  |  |  |  |  |  |  | 2.25 | 0.000 | 1.96 | 2.57 | 2.34 | 0.000 | 2.03 | 2.69 |
| Have you ever been diagnosed with kidney failure |  |  |  |  |  |  |  |  | 2.12 | 0.000 | 1.90 | 2.36 | 2.08 | 0.000 | 1.85 | 2.34 |
| Having families with psychosis |  |  |  |  |  |  |  |  | 2.37 | 0.000 | 2.19 | 2.57 | 2.34 | 0.000 | 2.15 | 2.54 |
| Have difficulty to access healthcare |  |  |  |  |  |  |  |  |  |  |  |  | 1.37 | 0.000 | 1.33 | 1.41 |
| Constant | 0.06 | 0.000 | 0.06 | 0.06 | 0.12 | 0.000 | 0.11 | 0.13 | 0.11 | 0.000 | 0.10 | 0.12 | 0.11 | 0.000 | 0.10 | 0.12 |

**Notes:** Model 1=adjusted with demographic and socioeconomics variables; Model 2=adjusted with demographic, socioeconomic, and lifestyle variables; Model 3=adjusted with demographic, socioeconomic, lifestyle and the presence of comorbidities variables; Model 4=adjusted with demographic, socioeconomic, lifestyle, the presence of comorbidities and access to healthcare variables. OR=Odds ratio; P=P-value; 95%CI=95% Confidence Intervals.

**Supplementary Figure 1** Marginal effect results with healthcare access among individuals living in (1) coastline areas with hazards and (2) outside coastline areas with hazards, which include those living in coastline without hazards and those living in non-coastline areas.


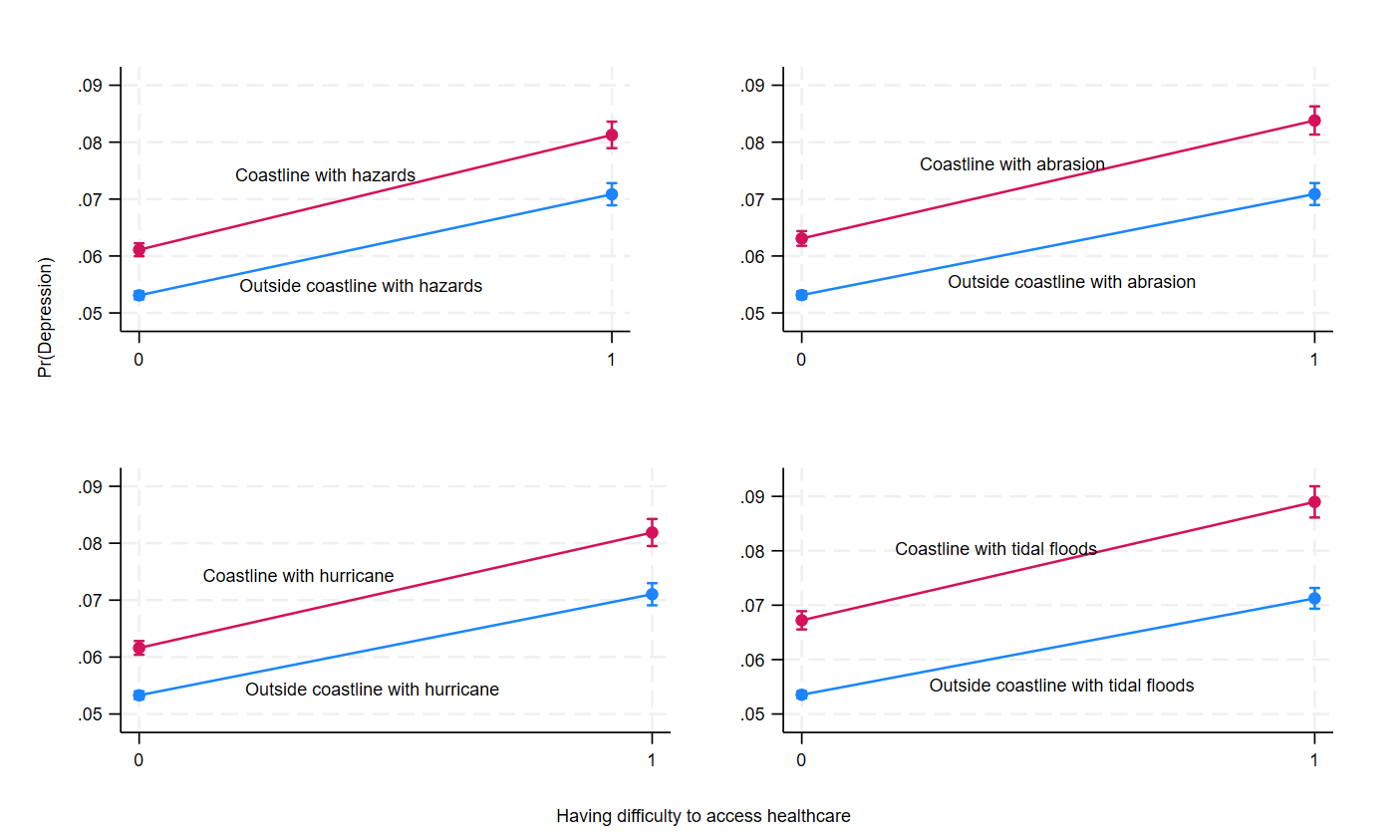


**Supplementary Figure 2** Marginal effect results with household expenditure among individuals living in (1) coastline areas with hazards and (2) outside coastline areas with hazards, which include those living in coastline without hazards and those living in non-coastline areas.


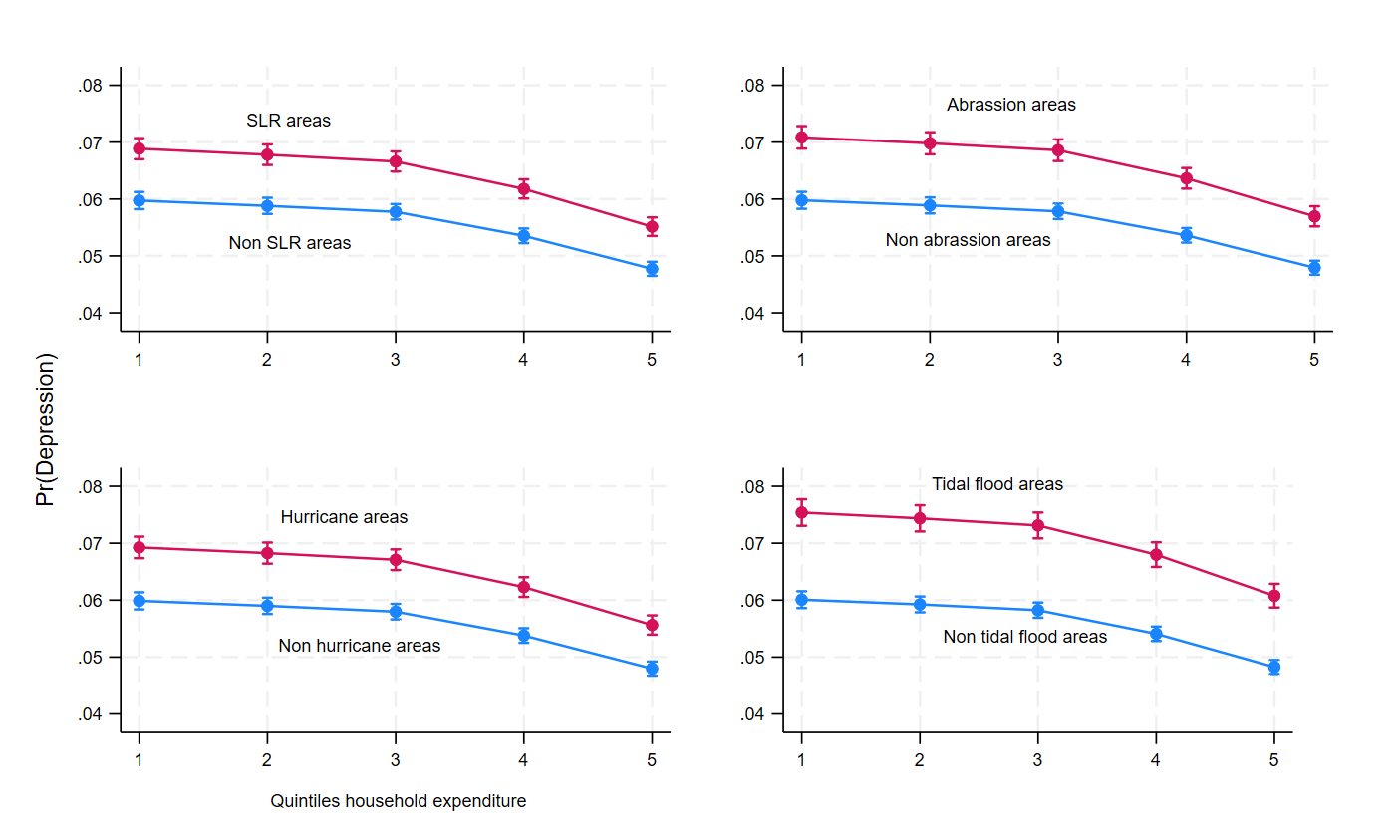

Supplement: Supplementary file 1 — Supplementary Material 1 [file 41598_2025_89298_MOESM1_ESM.docx]
